# The effect of pH on rates of reaction and hydrogen generation during serpentinization

**DOI:** 10.1098/rsta.2018.0428

**Published:** 2020-01-06

**Authors:** Thomas M. McCollom, Frieder Klein, Peter Solheid, Bruce Moskowitz

**Affiliations:** 1 Laboratory for Atmospheric and Space Physics, University of Colorado, Boulder, CO 80309, USA; 2 Marine Chemistry and Geochemistry Department, Woods Hole Oceanographic Institution, Woods Hole, MA 02543, USA; 3 Department of Earth Sciences, University of Minnesota, Minneapolis, MN 55455, USA; 4 Institute for Rock Magnetism, University of Minnesota, Minneapolis, MN 55455, USA

**Keywords:** serpentinization, hydrogen generation, subsurface life

## Abstract

A series of three laboratory experiments were conducted to investigate how pH affects reaction pathways and rates during serpentinization. Two experiments were conducted under strongly alkaline conditions using olivine as reactant at 200 and 230°C, and the results were compared with previous studies performed using the same reactants and methods at more neutral pH. For both experiments, higher pH resulted in more rapid serpentinization of the olivine and generation of larger amounts of H_2_ for comparable reaction times. Proportionally greater amounts of Fe were partitioned into brucite and chrysotile and less into magnetite in the experiments conducted at higher pH. In a third experiment, alkaline fluids were injected into an ongoing experiment containing olivine and orthopyroxene to raise the pH from circumneutral to strongly alkaline conditions. Increasing the pH of the olivine-orthopyroxene experiment resulted in an immediate and steep increase in H_2_ production, and led to far more extensive reaction of the primary minerals compared to a similar experiment conducted under more neutral conditions. The results suggest that the development of strongly alkaline conditions in actively serpentinizing systems promotes increased rates of reaction and H_2_ production, enhancing the flux of H_2_ available to support biological activity in these environments.

This article is part of a discussion meeting issue ‘Serpentinite in the Earth System’.

## 1. Introduction

Two of the defining characteristics of fluids discharged from low temperature (less than approx. 250°C), actively serpentinizing systems are their strongly alkaline pH and highly elevated H_2_ concentrations (e.g. [1–6]). The H_2_ in these fluids provides a source of metabolic energy to support lithoautotrophic microbial communities in alkaline springs on land and in serpentinite-hosted hydrothermal vents on the ocean floor [5–8]. Because the ultramafic rocks that undergo serpentinization are widespread in the solar system, similar processes may also generate H_2_ and support biological activity on other rocky planetary bodies [9–11]. Understanding the capacity of serpentinizing systems to support biological communities on Earth and beyond, therefore, requires knowledge of the flux of H_2_ that is generated as the reaction proceeds.

For this and other reasons, the serpentinization of ultramafic rocks has been the subject of progressively increasing numbers of laboratory experimental studies over the last few decades. Many of these studies have focused on understanding the impact that environmental variables such as temperature, rock composition, salinity and the activities of dissolved SiO_2_ and Al have on reaction rates and on the composition of products during serpentinization [12–28]. To date, however, there has been no systematic effort to study how pH affects serpentinization reactions. Nevertheless, the strongly alkaline conditions that prevail in many low-temperature serpentinizing environments suggests that the potential contribution of pH to reaction progress needs to be evaluated.

Previous experimental studies provide only limited information about the effect of strongly alkaline pH on serpentinization reactions. Lafay *et al*. [24] reacted olivine with extremely alkaline solutions (pH 13.5) and observed overall reaction rates that were much higher than observed in other recent studies of olivine serpentinization that were conducted at comparable temperatures and lower pH [12,13]. On the other hand, the rates observed by Lafay *et al*. were similar to those reported in other experimental studies [27,28], although a lack of information on the pH of the latter studies precludes evaluation of its possible role in higher reaction rates. In some other experiments, a transition from mildly alkaline to strongly alkaline pH during serpentinization was observed to be accompanied by a steep increase in H_2_ generation [14,19,25,26]. Taken together, these results suggest that higher pH may increase overall reaction rates and alter reaction pathway to enhance H_2_ production, but the role of pH during these experiments remains undetermined. By contrast to the results of serpentinization studies, laboratory experiments designed to monitor the dissolution of olivine or pyroxene have observed monotonically decreasing rates with increasing pH [29–32]. If the dissolution of the primary minerals is the rate-limiting step during serpentinization as suggested by some experimental studies [12,16], these observations would imply that the process should become slower with increasing pH.

In an effort to better understand the effect of pH on reaction pathways and rates during serpentinization, a series of three hydrothermal laboratory experiments were conducted for this study. The results reveal modified reaction pathways and increased rates of serpentinization and of H_2_ formation at high pH. We discuss possible mechanisms to account for observed pH effects and assess implications for natural systems undergoing serpentinization at moderate to low temperatures.

## 2. Methods

Three separate experiments were performed for this study, with initial ingredients and reaction parameters summarized in table 1. Two experiments were conducted by reacting olivine alone with strongly alkaline solutions at 200 and 230°C (Oliv200pH and Oliv230pH). Except for the elevated pH of the starting solution, the reactants and methods used in these experiments were designed to closely parallel previous olivine serpentinization experiments that attained a less strongly alkaline pH [13] (to facilitate comparison, the starting ingredients for the previous experiments are also listed in table 1). This parallel design allows for direct evaluation of the impact of pH on the reactions, since pH is essentially the only reaction parameter that varied between the experiments. The third experiment reacted a mixture of olivine and orthopyroxene (Opx) at 230°C (OlivOpx230pH). This experiment was allowed to react at 230°C and circumneutral pH for an extended period of time (approx. 4500 h). Additional fluid was then injected to raise the pH, and the experiment was allowed to continue to react for another 3200 h. Again, the reactants and methodology of this experiment were designed to closely parallel previous experiments with olivine–Opx mixtures as starting materials that developed a less alkaline pH as serpentinization proceeded [14].

**Table 1.** Summary of experimental parameters, with parameters used in comparable lower pH studies discussed in the text shown for reference. *m* = mmolal. Sources: (a) McCollom *et al*. [13] and (b) McCollom *et al*. [14].

experiment	source	temperature (°C)	mineral reactants	initial fluid (g)	fluid composition
Oliv230pH	this study	230	15.0 g olivine, <53 µm	45.8	480 *m* NaCl, 3 *m* Na_2_CO_3_, 33.8 *m* NaOH; pH 12.5
Oliv230fine	(a)	230	16.0 g olivine, <53 µm	39.5	485 *m* NaCl, 20 *m* NaHCO_3_; pH 7.8
Oliv200pH	this study	200	14.1 g olivine, 38–53 µm	51.2	483 *m* NaCl, 2 *m* Na_2_CO_3_, 32.9 *m* NaOH; pH 12.4
Oliv200fine	(a)	200	17.0 g olivine, 38–53 µm	46.2	486 *m* NaCl, 20 *m* NaHCO_3_; pH 6.4
OlivOpx230pH	this study	230	16.7 g olivine + 2.8 g Opx, 53–212 µm	35.8	486 *m* NaCl, 20 *m* NaHCO_3_; pH 7.8
OlivOpx230med	(b)	230	18.0 g olivine + 3.2 g Opx, 53–212 µm	45.4	485 *m* NaCl, 20 *m* NaHCO_3_; pH 7.8
OlivOpx230	(b)	230	21.0 g olivine + 3.5 g Opx, 53–212 µm	44.0	485 *m* NaCl, 20 *m* NaHCO_3_; pH 7.8

The experiments were conducted by heating powdered mineral substrates in the presence of aqueous solutions. The fluid composition was monitored during the experiments for the production of H_2_ and other dissolved compounds as the reactions proceeded. Solids were recovered at the end of the experiments and analysed for their mineral and chemical composition. Methods used to analyse the fluids and solid reaction products are summarized briefly below, with additional details provided as electronic supplementary material. All aspects of the experimental and analytical procedures are the same as those used in other recent studies [13,14].

The experiments were conducted in a flexible-cell hydrothermal apparatus using a gold reaction cell with titanium fittings [33]. The reaction cell was contained within a stainless steel pressure housing, with water used as the external pressurizing fluid. The flexibility of the gold reaction cell allowed fluids to be sampled from the reaction cell without loss of pressure and eliminated the presence of a vapour headspace so that reactions were confined to the aqueous phase. The cell was equipped with a capillary tube attached to a valve through which fluid samples were taken during the experiments in order to monitor reaction progress over time. The valve also allowed additional fluid to be injected into the reaction cell without disturbing the experiment. All titanium fittings that were exposed to reactants were combusted in air for at least 48 h at 400°C prior to use in the experiments in order to form a relatively inert TiO_2_ surface layer. Experiments were conducted at a pressure of 35 MPa.

The initial reactant fluid contained 485 mmol NaCl kg^−1^ to approximate seawater salinity, with NaOH added to increase the pH as appropriate. In order to monitor for the possible formation of hydrocarbons from the reduction of inorganic carbon [34], the experiments also included a source of dissolved inorganic carbon (DIC) in the form of either NaHCO_3_ or Na_2_CO_3_ at concentrations ranging from 2 to 20 mmol kg^−1^. The initial water : rock mass ratio ranged from 1.8 to 3.6, although the ratio within the reaction vessel decreased somewhat during the experiments as fluid aliquots were removed for chemical analysis and as water was incorporated into secondary phases.

The reactant minerals were pulverized by hand using a ceramic mortar and pestle, always avoiding the use of metal tools that could contaminate the reactants with metal particles that might generate H_2_ during heating. The resulting powders were sieved to achieve a defined range of grain sizes. For experiments using grains in the 38–53 µm or 53–212 µm size range, the sieved materials were washed repeatedly with deionized water (DI) to remove small particulates. The minerals used in the experiments were purchased from Excalibur Minerals (Charlottesville, VA, USA). The source of the olivine was mantle xenoliths from San Carlos, Arizona. Only olivine crystals larger than approximately 4 mm in diameter were used to prepare the mineral reactants, and partially crushed materials were hand-picked under a binocular microscope to exclude grains with obvious signs of weathering or inclusions of other minerals. Despite efforts to purify the reactant minerals, the olivine used in the experiments contained trace amounts (≪1 vol%) of chromian spinel, Opx and clinopyroxene. The Opx used in the experiments was from Bamble, Norway [35]. The prepared Opx reactant included less than 1% of several other contaminants including quartz, Ca-phosphate (probably chlorapatite), talc and albite. Magnetization analysis indicated the olivine and Opx prepared for the experiments contained 0.0001% and approximately 0.14 wt% magnetite, respectively. Chemical compositions of the reactant minerals are summarized in table 2.

**Table 2.** Chemical compositions of starting minerals and reaction products determined by electron microprobe analysis. Data for reaction products are average values (*n* = number of analyses) with standard deviation in parentheses.

oxide (wt%)	SC olivine	Bamble Opx	Oliv230pH chrysotile (*n* = 8)	Oliv230pH brucite (*n* = 3)	Oliv200pH chrysotile (*n* = 24)	Oliv200pH brucite (*n* = 14)	OlivOpx230pH chrysotile (*n* = 11)
SiO_2_	40.6	56.9	39.2 (1.5)	1.0 (0.6)	37.0 (1.9)	0.08 (0.01)	36.9 (2.7)
TiO_2_	<0.01	0.06	<0.05	<0.05	<0.05	<0.05	<0.05
Al_2_O_3_	0.03	0.14	1.8 (1.0)	<0.1	0.3 (0.4)	<0.05	1.3 (0.9)
FeO	8.9	9.5	4.8 (0.6)	22.3 (0.7)	3.1 (0.4)	16.3 (2.4)	7.8 (1.0)
MgO	50.1	32.8	35.6 (1.4)	43.3 (0.6)	34.0 (1.7)	47.8 (2.4)	30.0 (2.9)
MnO	0.14	0.08	<0.05	0.6 (0.1)	<0.05	0.47 (0.21)	0.07 (0.02)
CaO	0.07	0.28	<0.05	<0.05	<0.05	<0.05	—
Na_2_O	—	0.03	0.1	<0.05	<0.05	<0.05	—
K_2_O	—	<0.01	—	—	<0.05	<0.05	—
Cr_2_O_3_	<0.01	0.01	<0.05	<0.05	<0.05	<0.05	<0.05
NiO	0.38	0.02	0.22 (0.1)	0.18 (0.04)	0.21 (0.16)	<0.05	—
Cl	—	<0.01	0.16 (0.03)	0.37 (0.18)	—	0.69 (0.43)	—
total	100.1	100.4	82.0 (3.0)	67.8 (0.4)	75.0 (3.5)	74.2 (6.9)	76.0 (5.1)
Mg#	91	86	93 (0.9)	78 (0.7)	95 (0.7)	89 (1.0)	87 (2.2)
(Mg + Fe) : Si^a^			1.45		1.43		1.39

^a^Molar ratio.

At intervals throughout the experiments, several fluid aliquots (0.3–1 g each) were taken directly into glass gas-tight syringes and analysed for dissolved volatile species including H_2_, total dissolved ${\text{CO}}_{2}(\sum {\text{CO}}_{2}={\text{CO}}_{2(\text{aq})}+{\text{HCO}}_{3}^{-}+{{\text{CO}}_{3}}^{2-}$), CH_4_ and C_2_–C_6_ hydrocarbons. These compounds were measured by gas chromatography (GC) with thermoconductivity detection (TCD) or flame ionization detection (FID) following a headspace extraction. In most cases, the reported H_2_ concentrations are averages of three to four separate fluid aliquots, while other dissolved gases were typically only measured for a single aliquot to conserve fluid. For comparative purposes, the measured H_2_ concentrations were normalized to the total number of moles generated per gram of reactant minerals to account for variations in the amounts of solids used in different experiments and for the changing mass of fluid present in the reaction vessel as aliquots are removed during sampling. Only trace amounts (less than 1 µmol kg^−1^) of C_2_–C_6_ hydrocarbons were observed in any of the experiments, so individual results are not reported. Estimated errors for volatile analyses are ±5%. Fluid aliquots remaining after extraction of volatiles were used for measurements of pH, total dissolved SiO_2_ (ΣSiO_2_) and major elements. Detailed methods for fluid analyses are provided in the electronic supplemental material.

At the termination of the experiments, the solids were removed, filtered and rinsed with DI water. The solids were characterized by a variety of methods including scanning electron microscopy (SEM) coupled with energy-dispersive X-ray spectroscopy (EDS), X-ray diffraction (XRD), electron microprobe analysis (EMPA), confocal Raman spectroscopy, thermogravimetric analysis (TGA) and magnetic methods (see electronic supplementary material for details). To obtain additional information on the distribution of Fe among secondary phases, Mössbauer spectroscopy (MB) was also performed on the reaction products.

Thermodynamic calculations were conducted to determine fluid speciation, estimate *in situ* pH at experimental conditions, and construct mineral stability diagrams. The calculations used the 35 MPa database described by McCollom & Bach [36], which is based on thermodynamic data from Helgeson *et al*. [37], Shock & Helgeson [38] and Shock *et al*. [39,40]. Thermodynamic properties for solid solutions were calculated from end-member data assuming ideal mixing. Fluid speciation calculations to estimate *in situ* pH and silica activity were performed in two steps using the computer program EQ3 [41]. First, the fluid was speciated at 25°C using the measured fluid compositions, including the room temperature pH, and adjusted for charge balance with Na. The total dissolved Na calculated at 25°C was then used with other measured concentrations to respeciate the fluid at the reaction temperature, with charge balance determining the *in situ* pH. In those cases where Mg, Ca and Fe were below detection limits, the total dissolved abundance of these elements were assumed to be 1 µmol kg^−1^, 1 µmol kg^−1^, 0.1 µmol kg^−1^, respectively. Mineral stability diagrams were constructed using the computer program Geochemist's Workbench (Aqueous Solutions LLC, Champaign, IL, USA).

## 3. Results

The results of individual experiments are summarized in the following subsections. Key observations from the experiments are summarized in table 3. Selected components of the fluids are shown in the accompanying figures, along with fluid compositions from analogous experiments from previous studies performed under circumneutral to mildly alkaline conditions. A table providing more complete measured fluid compositions for experiments from the present study is included in the electronic supplementary material, table S1. Analyses of solid products by XRD are shown in electronic supplementary material, figure S1, and are incorporated into the summaries below.

**Table 3.** Summary of experimental results from this and previous studies. Numbers in parentheses are standard deviations. The *in situ* pH is calculated from the measured room temperature pH as described in the methods section. ‘—’, not determined or not present; b.d., below detection; Srp, serpentine; Brc, brucite; Mgt, magnetite.

							Mg#			
experiment	temperature (°C)	duration (h)	final pH_25°C_	final pH*_in situ_*	final H_2_ conc. (mmol kg^−1^)	total H_2_ generated (μmol g^−1^ minerals)	srp.	brc.	Wt% mgt.	estimated wt% reaction
Oliv230pH	230	2972	12.3	9.3	47	105	93 (0.9)	78 (1)	1.6	70
Oliv230fine^a^	230	4293	9.1	7.8	25	47	97 (0.4)	92 (1.2)	1.75	25
Oliv200pH	200	7677	11.3	8.5	8.4	20.9	95 (0.7)	84 (1.8)	1.0	41
Oliv200fine^a^	200	9219	9.2	8.2	2.9	5.9	95 (0.8)	91 (1.1)	0.25	5.9
OlivOpx230pH	230	7715	10.3	8.7	7.4	13.5	88 (1.4)	—	0.02	17
OlivOpx230med^b^	230	2709	7.2	7.8	0.008	0.016	—	—	b.d.	1.9
OlivOpx230^b^	230	9287	11.7	8.8	61.0	90.7	91 (0.9)	87 (1.7)	1.52	53

^a^McCollom *et al*. [13]; note that values for estimated wt% reaction are slightly lower than originally reported owing to an error discovered in the calculations.

^b^McCollom *et al*. [14].

### (a) Reaction of olivine at 230°C

**Figure 1.**
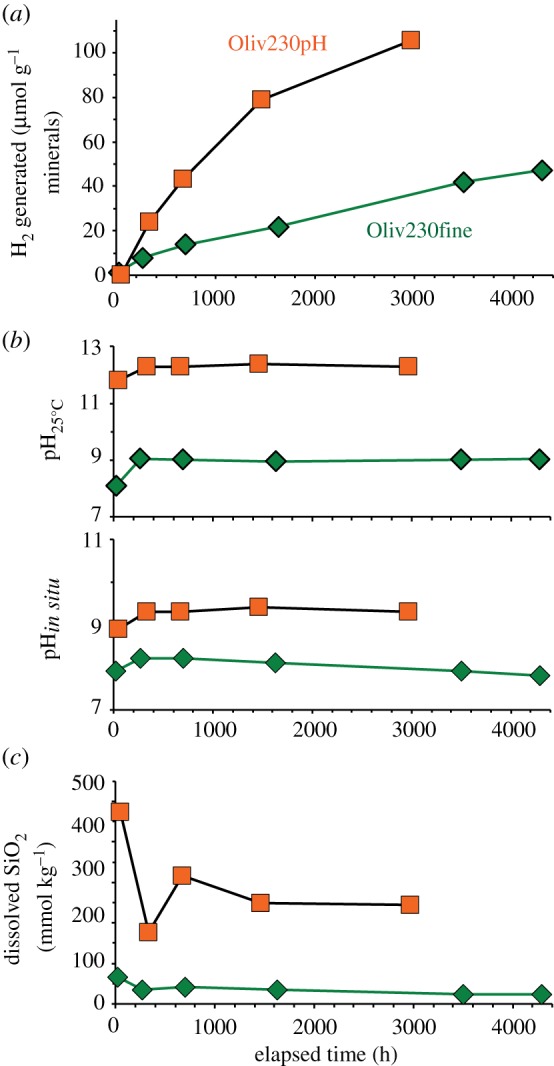
Fluid compositions during olivine experiment Oliv230pH (squares), with results for Oliv230fine (diamonds) from McCollom *et al*. [13] shown for comparison. (*a*) Total amount of H_2_ generated per gram mineral reactant. (*b*) Measured room temperature pH (pH_25°C_) and calculated *in situ* pH (pH*_in situ_*). (c) Total dissolved SiO_2_ concentration. (Online version in colour.)

Experiment Oliv230pH was conducted to evaluate the reaction of olivine with a strongly alkaline pH solution at 230°C (table 1). The temperature and initial reactants for this experiment were essentially the same as those used in experiment Oliv230fine reported in McCollom *et al*. [13] (table 1), except that the initial pH of the reactant fluid for Oliv230pH was increased to 12.4 by the addition of NaOH (versus an initial pH of 7.8 in Oliv230fine). The amount of DIC added was also reduced (from 20 mmol kg^−1^ to 3 mmol kg^−1^) to limit the pH buffering capacity of the fluid and the precipitation of carbonate minerals. Measured compositions of selected components in the fluid during Oliv230pH are shown in figure 1, with fluid compositions during Oliv230fine shown for comparison.

The room temperature pH (pH_25°C_) of Oliv230pH was 11.8 after 2 days of heating, but rose to 12.3 by the time of the second sample at 333 h and then remained near this level for the remainder of the experiment (figure 1*b*). The calculated pH at experimental conditions (pH*_in situ_*) reached 9.3 after 333 h and remained at this level throughout the experiment, reflecting strongly alkaline conditions (figure 1*b*; for reference, neutral pH at the temperature of the experiment is about 5.5). In experiment Oliv230fine without the added NaOH, the pH_25°C_ was near 8.9 at the start of the experiment, and levelled off at around 9 for the remainder of the experiment. The calculated pH*_in situ_* for Olive230fine rose to 8.2 after 262 h, and then slowly declined to a final value of 7.8 at the termination of the experiment (figure 1*b*).

Generation of H_2_ in Oliv230pH began immediately upon heating and continued throughout the experiment, although the rate appeared to taper off slightly as the reaction progressed (figure 1*a*). By the end of the experiment, a total of 105 µmol (g olivine)^−1^ of H_2_ had been produced (note that here and elsewhere the amount of H_2_ generated is indexed to the mass of the original amount of mineral reactant, not the proportion of minerals that had reacted). The generation of H_2_ also began immediately in Oliv230fine and increased steadily during the experiment. However, rates of H_2_ generation in Oliv230pH were about three times higher than was observed for Oliv230fine (figure 1*a*).

The concentration of total dissolved silica (ΣSiO_2_) was relatively high (480 µmol kg^−1^) at the start of Oliv230pH, and decreased to lower levels as the experiment progressed to attain a final concentration of 240 µmol kg^−1^ (figure 1*c*). The ΣSiO_2_ concentration at 333 h was significantly lower than other measurements during this experiment; however, it is not clear whether this represents an actual dip in concentration or a spurious measurement. In any case, ΣSiO_2_ concentrations throughout Oliv230pH remained well above those observed in the lower pH Oliv230fine experiment (less than or equal to 43 µmol kg^−1^; figure 1*c*). The concentration of Na in the fluid from Oliv230pH remained constant during the experiment, and most other cations (Mg, Fe, Ca, Al) were present at levels below a few micromoles per kilogram (electronic supplementary material, table S1). However, significant amounts of dissolved K at approximately 7 mmol kg^−1^ were observed in all samples. The source of K for the experiments is uncertain, but is likely an unidentified trace mineral present in the initial reactants. Similar concentrations of cations were observed during Oliv230fine, although K was much lower at approximately 0.18 mmol kg^−1^.

**Figure 2.**
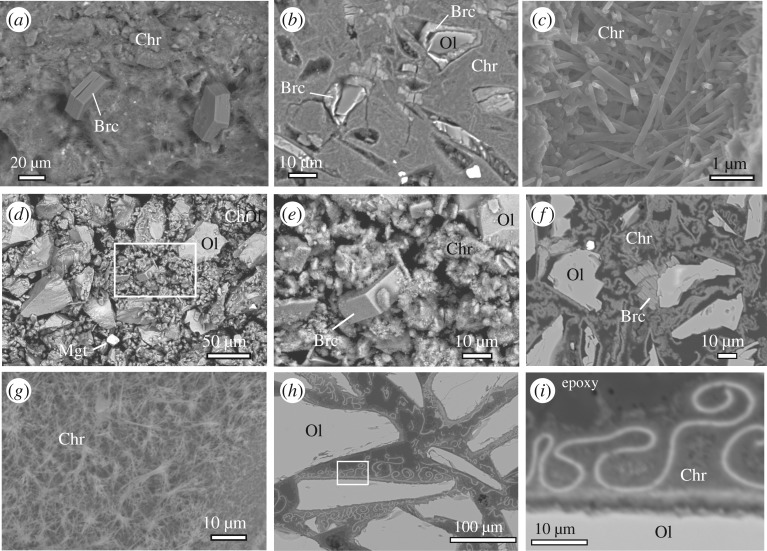
Back-scattered electron images of representative reaction products from experiments: (*a*–*c*) Oliv230pH, (*d*–*f*) Oliv200pH and (*g*–*i*) OlivOpx230pH. Images (*b*, *f*, *h* and *i*) show polished cross-sections of solids embedded in epoxy, others are from particles mounted on aluminium stubs. Bright spots are magnetite. (*a*) Thick mat of fibrous chrysotile with embedded brucite deposited on top of olivine. (*b*) In cross-section, chrysotile from Oliv230pH forms thick accumulations that cement sample together as a solid mass; void spaces indicate places where remnant olivine or brucite have been removed during sample preparation. (*c*) Chrysotile fibres. (*d*) Box outlines area shown expanded in image (*e*). (*g*) Mat of chrysotile fibres deposited on olivine. (*h*) Box outlines area shown expanded in image (*i*).

Secondary mineral products of Oliv230pH were dominated by chrysotile and brucite, along with minor magnetite and trace amounts of calcite (figure 2; electronic supplementary material, figure S1). Also present scattered among the products were very fine-grained particles with elevated Ni and Fe contents as indicated by EDS analysis (electronic supplementary material, figure S2). The lack of elevated amounts of S or O associated with these particles suggests that they likely consist of awaruite (nominally Ni_3_Fe). In addition to these secondary products, a small fraction of the original olivine remained at the termination of the experiment (table 3).

The chrysotile formed thick fibrous accumulations, while the brucite was generally present as platy hexagonal crystals embedded in the chrysotile (figure 2*a,c*). Although the original reactants were powders, the chrysotile fibres cemented the products into a solid mass with limited porosity (figure 2*b*). In some cases, the brucite was found in close contact with remnant olivine (figure 2*b*). The chrysotile has a Mg# [ = 100 × Mg/(Mg + Fe), molar basis] equal to 93, while brucite has a Mg# of 78 (table 2). No spatial variation in the chemical composition of these minerals was observed. Analyses of brucite crystals by EMPA and EDS showed that it contains up to 2 wt% Cl, indicating the brucite is intergrown with iowaite as observed in previous studies [13,14]. For both chrysotile and brucite, the minerals are more enriched in Fe (i.e. lower Mg#) than was observed for Oliv230fine (table 3), which reflects favoured partitioning of Fe into chrysotile and brucite in the higher pH experiment. The products of Oliv230pH contained 1.60 wt% magnetite versus 1.75 wt% in Oliv230fine.

Mössbasuer analysis of the secondary products from Oliv230pH indicated the presence of four doublets, with two peaks assigned each to Fe^II^ and Fe^III^ (table 4). The three main doublets (Fe^II^ and Fe^III^) had hyperfine parameters consistent with chrysotile, with the occupation of both octahedral and tetrahedral sites by the Fe^III^ [42]. Adding a second ferrous doublet (assigned to brucite) slightly improved the fit, but only accounts for 8% of the total spectral area. The Fe^II^ doublets for brucite and chrysotile overlap making it difficult to resolve these two phases in the MB spectra, particularly when brucite is subordinate. However, because brucite was identified in this sample by other methods, fitting with two ferrous doublets was justified. Note that the MB analyses for experiments in this study were performed on secondary products that had been physically separated from remnant reactant minerals by sonication and suspension in ethanol, so there is no doublet for remnant olivine (see electronic supplementary material).

**Table 4.** Room temperature hyperfine magnetic Mössbauer parameters for experimental samples and reactant minerals.

sample	QS (mm s^−1^)	IS (mm s^−1^)	%^a^	assignment
Oliv230pH	2.75	1.08	43	Fe^II^ (chrysotile)
	0.81	0.42	31	Fe^III^ (chrysotile, oct)
	0.66	0.20	18	Fe^III^ (chrysotile, tet)
	2.79	1.22	8	Fe^II^ (brucite)
Oliv200pH	2.81	1.10	43	Fe^II^ (chrysotile)
	0.75	0.38	28	Fe^III^ (chrysotile, oct)
	0.47	0.18	17	Fe^III^ (chrysotile, tet)
	2.84	1.24	12	Fe^II^ (brucite)
Oliv200fine^b^	2.90	1.17	86	Fe^II^ (chrysotile)
	0.58	0.29	14	Fe^III^ (chrysotile, oct)
OlivOpx230pH	2.72	1.11	71	Fe^II^ (chrysotile)
	0.86	0.43	14	Fe^III^ (chrysotile, oct)
	0.52	0.24	15	Fe^III^ (chrysotile, tet)
SC olivine	3.11	1.14	55	Fe^II^
	2.87	1.14	45	Fe^II^
Bamble Opx	2.05	1.13	51	Fe^II^ (M2)
	1.81	1.00	49	Fe^II^ (M1)

^a^Relative molar abundances of Fe^II^ and Fe^III^ among the identified components. Peak assignments for Fe^III^ in serpentine minerals correspond to either octahedral (oct) or tetrahedral (tet) sites.

^b^McCollom *et al*. [13].

**Figure 3.**
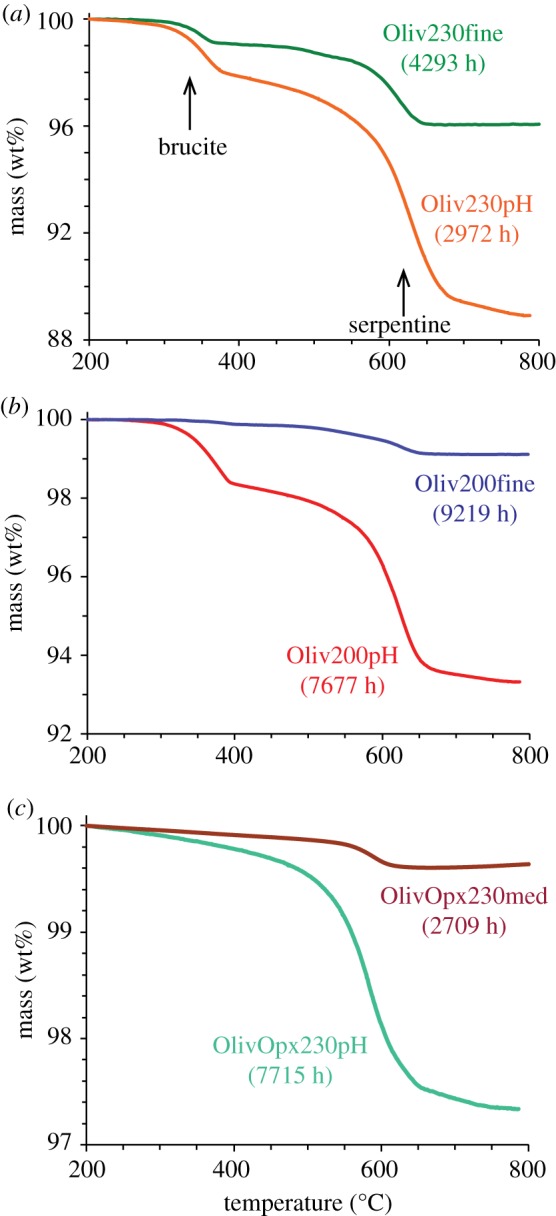
Results of thermogravimetric analysis of reacted solids for laboratory experiments, with results from previous studies shown for comparison. (*a*) Olivine experiments at 230°C, (*b*) olivine experiments at 200°C, and (*c*) mixed olivine-Opx experiments at 230°C. Mass loss between 250 and 450°C is assigned to brucite and that for 500–750°C is assigned to serpentine (chrysotile). (Online version in colour.)

Thermogravimetry of Oliv230pH resulted in mass losses from the decomposition of chrysotile and brucite (figure 3). About 8.1 wt% mass loss is attributable to dehydration of chrysotile while 2.3 wt% is attributable to brucite, which translates to a molar chrysotile : brucite ratio of approximately 1.8 (table 5). Based on the TGA results, approximately 70 wt% of the olivine had reacted by the time the experiment was terminated (table 5). This is a substantially greater amount of reaction progress than was observed for Oliv230fine (approx. 25 wt% olivine reacted) despite the considerably longer reaction time for that experiment (4993 h for Oliv230fine versus 2972 h for Oliv230pH).

**Table 5.** Results of thermogravimetric analyses for experiments from this study and for comparable experiments at lower pH from previous studies.

	Oliv230pH	Oliv230fine^a^	Oliv200pH	Oliv200fine^a^	OlivOpx230pH	OlivOpx230med^b^
TGA weight loss (wt%)						
serpentine	8.1	3.0	4.7	0.75	2.3	0.27
brucite	2.3	0.9	1.9	0.15	0	0
secondary minerals (wt%)						
serpentine	64.0	24.0	37.0	6.0	18.6	2.2
brucite	8.0	3.1	6.7	0.51	0	0
magnetite^c^	1.6	1.75	1.0	0.25	0.02	0
extent of reaction (%)^d^	70	25	41	5.9	19	1.9
serpentine : brucite (molar ratio)	1.76	1.65	1.24	2.5	—	—

^a^McCollom *et al*. [13].

^b^McCollom *et al*. [14].

^c^From magnetization measurements.

^d^Estimated percent of total primary minerals reacted, by weight.

### (b) Reaction of olivine at 200°C

**Figure 4.**
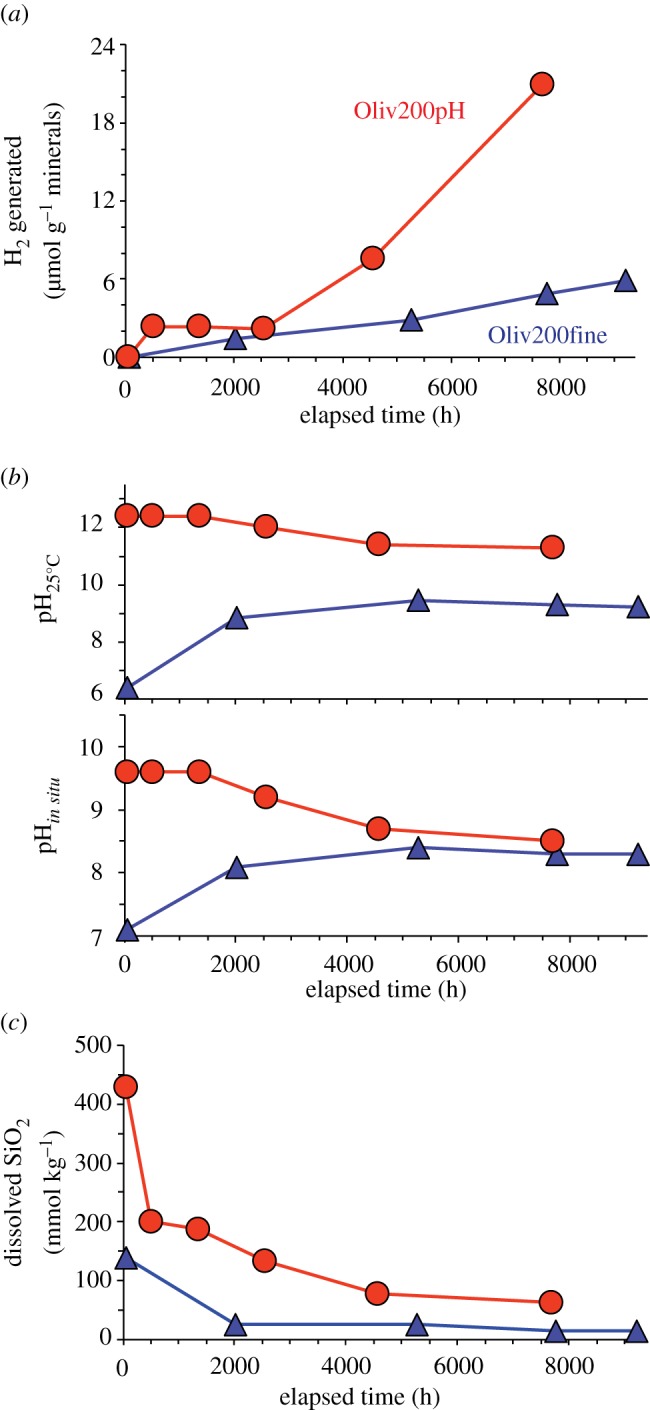
Fluid compositions during olivine experiment Oliv200pH (circles), with results for Oliv200fine (triangles) from McCollom *et al*. [13] shown for comparison. (*a*) Total amount of H_2_ generated per gram mineral reactant. (*b*) Measured room temperature pH (pH_25°C_) and calculated *in situ* pH (pH*_in situ_*). (*c*) Total dissolved SiO_2_ concentration. (Online version in colour.)

Experiment Oliv200pH was conducted to evaluate the reaction of olivine in the presence of strongly alkaline pH at 200°C (table 1). Initial conditions for this experiment were designed to parallel those of Oliv200fine from McCollom *et al*. [13] except for the addition of NaOH to raise the initial pH and inclusion of a smaller amount of DIC. Measured concentrations of selected dissolved components in the fluid during this experiment are shown in figure 4, with measurements of the same components from Oliv200fine shown for comparison.

The pH_25°C_ in Oliv200pH was initially 12.4 and remained at this level for around 1300 h before gradually decreasing to a final value of 11.3 (figure 4*b*). The calculated pH*_in situ_* was 9.6 at the first measurement and decreased over time to a final value of 8.5. Generation of H_2_ in Oliv200pH began soon after heating and the amount produced had risen to 2.3 µmol (g olivine)^−1^ by the time of the second measurement at 502 h (figure 4*a*). However, the H_2_ abundance in the fluid then decreased slightly over the next approximately 2000 h before increasing once again to reach a final amount of 20.9 µmol (g olivine)^−1^ at 7677 h when the experiment was terminated (figure 4*a*). For comparison, the measured pH_25°C_ in Oliv200fine at the first measurement was 6.4, but then increased to 8.8 by the second sample at 2018 h and remained near this value for the remainder of the experiment (figure 4*b*). The corresponding pH*_in situ_* was initially 7.1 but then rose to level off at values around 8.3, similar to the pH*_in situ_* in the latter stages of Oliv200pH. The amount of H_2_ generated in Oliv200fine increased steadily over time to a final value of 5.9 µmol (g olivine)^−1^ at 9219 h, less than 30% of the amount produced in Oliv200pH (figure 4*a*).

**Figure 5.**
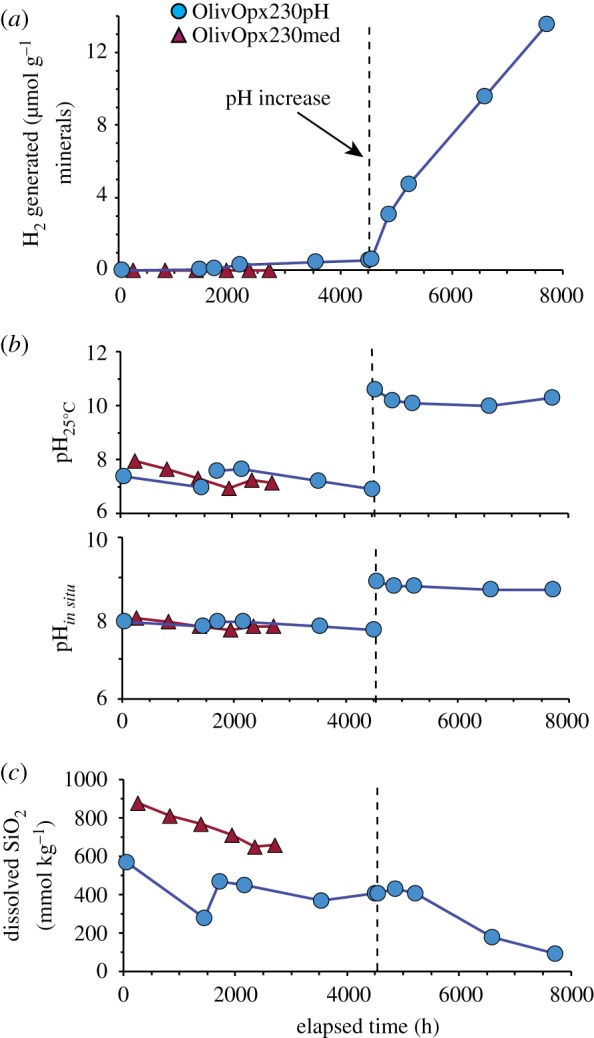
Fluid compositions during olivine experiment OlivOpx230pH (circles), with results for OlivOpx230med (triangles) from McCollom *et al*. [14] shown for comparison. The vertical dashed lines denote the timepoint of pH increase induced by the injection of a strongly alkaline solution. (*a*) Total amount of H_2_ generated per gram mineral reactant. (*b*) Measured room temperature pH (pH_25°C_) and calculated *in situ* pH (pH*_in situ_*). (*c*) Total dissolved SiO_2_ concentration. (Online version in colour.)

The dissolved silica concentration was relatively high (430 µmol kg^−1^) at the beginning of Oliv200pH, but decreased to 200 µmol kg^−1^ in the first 500 h and then continued to decrease steadily as the experiment progressed to attain a final concentration of 60 µmol kg^−1^ (figure 4*c*). Concentrations of ΣSiO_2_ during the latter stages of Oliv200pH converged on those in Oliv200fine (10–30 µmol kg^−1^), but remained slightly higher. Measured concentrations of K in Olvi200pH ranged from 500 to 1000 µmol kg^−1^ during the experiments, while concentrations of other cations (Mg, Ca, Fe, Al) remained at very low levels (70 µmol kg^−1^ or less; electronic supplementary material, table S1). Similar cation abundances were observed in Oliv200fine.

Chrysotile and brucite were the predominant secondary mineral products of Oliv200pH, along with magnetite and trace calcite (figure 2; electronic supplementary material, figure S1). A substantial fraction of the original olivine also remained at the end of the experiment. The morphology of chrysotile and brucite were very similar to the products of Oliv230pH (figure 2*d–f*). The chrysotile in Oliv200pH had the same chemical composition (Mg# = 95) as in Oliv200fine, but brucite had higher Fe contents in Oliv200pH (Mg# = 84) than Oliv200fine (Mg# = 91; table 3). As in other similar experiments, the brucite in Oliv200pH contained substantial amounts of Cl indicating it was intergrown with iowaite [13,14]. Magnetization measurements indicated that 1.0 wt% magnetite was produced during Oliv200pH, substantially more than in Oliv200fine (0.25 wt%).

Mössbasuer analysis of the products from Oliv200pH identified three main doublets, with one peak assigned to Fe^II^ and two peaks to Fe^III^ (table 4). The hyperfine parameters for all three peaks are consistent with chrysotile, with the occupation of both octahedral and tetrahedral sites by the Fe^III^. Similar to the Oliv230pH results, adding another Fe^II^ doublet assigned to brucite (12 mol% of total Fe) slightly improved the final fit. Approximately equal amounts of Fe^II^ and Fe^III^ are present in the chrysotile, with about two-thirds of the Fe^III^ occupying the octahedral site. Thermogravimetry of Oliv200pH showed significant weight loss attributable to dehydration of both chrysotile (4.7 wt%) and brucite (1.9 wt%) corresponding to a chrysotile : brucite mass ratio of 1.2, with substantially greater amounts of both products than in Oliv200fine (figure 3; table 3). The proportion of olivine reacted in Oliv200pH was about 41 wt%, much greater than in Oliv200fine (5.9 wt%).

### (c) Reaction of olivine–Opx mixture at 230°C

Experiment OlivOpx230pH was performed with a mixture of olivine and Opx as reactants at 230°C, and involved increasing the pH during the experiment from circumneutral to strongly alkaline conditions (table 1). The reactants for this experiment were designed to parallel those used in a previous series of experiments with olivine–Opx mixtures performed under the same conditions but without externally adjusting the pH [14]. Fluid compositions during the experiment are shown in figure 5, along with the fluid composition from a previous experiment using essentially the same initial ingredients but performed for a somewhat shorter period of time at circumneutral pH (OlivOpx230med; table 1).

The initial reactant fluid for OlivOpx230pH had a circumneutral pH, and the measured pH_25°C_ in the experiment after 52 h of heating was 7.4 (figure 5*b*). A first attempt to increase the pH was made after 1530 h, when a fluid with pH_25°C_ = 10.6 was injected into the reaction cell (electronic supplementary material, table S1). However, analysis of the fluid approximately 200 h after the injection indicated that the pH_25°C_ had risen only slightly to 7.6, indicating the pH of the fluid had been buffered by reaction with the solids in the reaction cell. The experiment was then allowed to continue to react at circumneutral pH for another 2800 h. Throughout this initial stage of the experiment, the pH_25°C_ of OlivOpx230pH remained at levels very similar to OlivOpx230med (figure 5*b*). A second attempt to increase pH was made at 4526 h, when a fluid with pH_25°C_ = 12.3 was injected (electronic supplementary material, table S1). This injection successfully raised the pH_25°C_ of the fluid in the reaction cell to 10.6, and it remained above 10 for the remainder of the experiment (figure 5*b*).

Very little production of H_2_ was observed in OlivOpx230pH during the first 4503 h of the experiment prior to the increase of pH, although there was a very slight increase in the rate of H_2_ accumulation following the first injection at 1530 h (figure 5*a*). After the increase in pH, however, there was a steep increase in H_2_ production that continued at a steady rate for the remainder of the experiment (figure 5*a*). By the end of the experiment, 13.5 µmol of H_2_ had been generated per gram of reactant minerals (figure 5*a*). Virtually no H_2_ production was observed in the previous OlivOpx230med experiment at a lower pH.

The ΣSiO_2_ concentration in OlivOpx230pH remained fairly steady at around 400 µmol kg^−1^ up until the time of the pH increase, with concentrations slightly lower than those observed in OlivOpx230med (figure 5*c*). Shortly after the pH increase, the ΣSiO_2_ concentration in OlivOpx230pH began to steadily decrease and reached 100 µmol kg^−1^ at the termination of the experiment. Dissolved Ca, Mg and Al were observed in the early stages of OlivOpx230pH at concentrations of 50–100 µmol kg^−1^, but only Ca persisted at levels above the detection limit after the pH increase (electronic supplementary material, table S1). Dissolved K was present at around 200 µmol kg^−1^ before the pH increase but fell below detection afterwards, while Fe was below detection throughout the experiment. Concentrations of Na and Cl remained constant within analytical errors throughout the experiment. Calcium was the only cation detected other than Na in OlivOpx230med, with a final concentration of 60–100 µmol kg^−1^.

The secondary reaction products of OlivOpx230pH were dominated by chrysotile (figure 2*g*–*i*). The chrysotile fibres formed thin mats that covered the surfaces of olivine and Opx crystals, which curled up into the void spaces between the reactant minerals (figure 2*i*). The solid reaction products also included trace amounts of magnetite and calcite. Both relict olivine and Opx remained at the end of the experiment; however, analysis of the reacted solids by XRD (electronic supplementary material, figure S1) showed that the olivine : Opx ratio was far higher in the products than in the unaltered reactants, and examination of the products by SEM detected very few remnants of unreacted Opx. Therefore, Opx evidently reacted more extensively than olivine during the experiment. In the experiment OlivOpx230med at lower pH, only a very limited amount of secondary products was observed, forming a very thin rind on the surfaces of olivine and Opx crystals [14]. Alteration products in the rind from OlivOPx230med were poorly crystalline, but were composed of a mixture of serpentine (most likely lizardite) and minor amounts of talc.

The chrysotile that formed in OlivOpx230pH had an Mg# = 87, intermediate between the Mg#s of the original olivine and Opx (table 2). Mössbauer analysis indicates that approximately 29 mol% of the total Fe in the serpentine was present as Fe^III^, with nearly equal amounts occurring in octahedral and tetrahedral coordination (table 4). The reacted solids included only 0.02 wt% magnetite. Since this is about the same amount that would have been present in the reactants from Opx, it is not clear if any of this magnetite was produced during the experiment. Weight loss during TGA was entirely attributable to dehydration of serpentine (2.3 wt%), corresponding to reaction of about 19 wt% of the original mineral reactants (figure 4; table 3). Only 1.9 wt% of the original minerals had reacted in OlivOpx230med, which lasted only approximately 35% as long as OlivOpx230pH.

## 4. Discussion

### (a) Effect of pH on reaction of olivine

Both of the olivine serpentinization experiments performed for this study show evidence for steep increases in overall reaction rates and the rate of H_2_ production at higher pH. At 230°C, an increase in pH*_in situ_* of approximately 1.2 units between Oliv230pH and Oliv230fine resulted in a nearly threefold increase in the extent of olivine reaction and greater than twofold increase in the amount of H_2_ generated, despite the significantly shorter reaction time for Oliv230pH (table 3). At 200°C, the pH*_in situ_* of Oliv200pH and Oliv200fine converged as the reactions progressed, yet the extent of olivine reaction was still about seven times greater in Oliv200pH and more than three times as much H_2_ was generated.

To a large extent, the greater rate of H_2_ production in the higher pH experiments can be attributed to the increased overall rate of olivine reaction. However, in both of the high pH olivine experiments, the increase in the amount of H_2_ generated is less than the increase in the extent of reaction (table 3). Since generation of H_2_ during serpentinization is linked to the conversion (oxidation) of ferrous Fe (Fe^II^) from the primary minerals to ferric Fe (Fe^III^) in the secondary mineral products [36,43], the lower H_2_ yields as a function of reaction progress in the higher pH experiments must reflect changes in the distribution of Fe among the solid products.

Analyses of mineral compositions show significant differences in how Fe is distributed among the reaction products in the high pH experiments relative to their lower pH counterparts (table 3). Despite a much greater overall extent of reaction, less magnetite was produced during Oliv230pH (1.6 wt%) than in Oliv230fine (1.75 wt%). While more magnetite was produced during Oliv200pH (1.0 wt%) than in Oliv200fine (0.25 wt%), the amount produced relative to the proportion of olivine reacted was substantially less in Oliv200pH. The Fe content of brucite was significantly greater in both of the high pH experiments relative to their lower pH counterparts, and the Fe content of chrysotile was also greater in Oliv230pH (table 3). Furthermore, the Fe^III ^: Fe_total_ ratio in chrysotile was substantially higher in Oliv230pH and Oliv200pH (0.53 and 0.51, respectively) than in lower pH olivine experiments, where the ratio for chrysotile is consistently in the 0.14–0.24 range (table 4; [13]).

These observations indicate that the higher pH experiments resulted in more Fe being partitioned into brucite and chrysotile and less into magnetite from each increment of olivine reacted. At the same time, a greater proportion of Fe^III^ was partitioned into chrysotile rather than into magnetite. The ultimate outcome was that each increment of olivine that underwent serpentinization generated less H_2_ in the higher pH experiments, with proportionally less of the H_2_ derived from Fe^III^ partitioned into magnetite and more from Fe^III^ precipitated as chrysotile.

Further study is required to determine the mechanisms that caused the changes in the partitioning of Fe among the reaction products at higher pH. One possibility, however, is that higher pH may alter the speciation of dissolved components in ways that effect the relative rates of mineral precipitation and uptake of Fe species. For instance, higher ΣSiO_2_ and OH^−^ concentrations may have allowed more rapid precipitation and uptake of Fe by chrysotile and brucite, and slowed precipitation of magnetite. Another possibility is that the higher pH altered the surface charge of precipitating minerals and allowed Fe to be more efficiently taken up by chrysotile and brucite. Additionally, at higher pH, a greater proportion of the dissolved Fe would be present as hydroxide complexes such as FeOH^+^ (e.g. [26]), and such positively charged species may more readily interact with the surfaces of precipitating brucite and lead to more rapid uptake.

Other investigators have suggested that the greater proportion of FeOH^+^ at higher pH may also lead to increased H_2_ generation and magnetite precipitation owing to faster rates of oxidation of FeOH^+^ relative to other dissolved Fe species [26,44]. For instance, Syverson *et al*. [26] observed a substantial increase in the rate of H_2_ generation following an increase in pH during the reaction of an olivine–talc mixture at 300°C, and attributed the higher rate of H_2_ generation to a faster rate of Fe oxidation and magnetite precipitation following the change in pH. However, the results of the present study suggest that much of the increase in H_2_ generation in their experiment might instead be attributable to an increase in the overall rate of serpentinization of olivine following the increase in pH rather than to a change in Fe speciation (as in this study, Syverson *et al*. only examined the mineral products at the termination of the experiments, so the possibility of changes in reaction rates during their experiment cannot be directly evaluated). Furthermore, rather than enhancing magnetite precipitation as inferred by Syverson *et al*. [26], the results of the present study suggest that higher pH may actually favour precipitation of chrysotile and brucite relative to magnetite. Nevertheless, it is possible that changes in Fe speciation may have affected to some degree the rate of oxidation and resulting distribution of Fe^III^ between magnetite and chrysotile in the present set of experiments.

The distribution of Fe among product minerals may also have been influenced by thermodynamic factors. For example, thermodynamic equilibrium favours greater partitioning of Fe into brucite in coexistence with magnetite as H_2_ activities increase, as reflected in the generalized reaction

4.1 $$3{\left[\text{Fe}{\left(\text{OH}\right)}_{2}\right]}_{\text{brucite}}↔\text{ F}{\text{e}}_{3}{\text{O}}_{4}[\text{magnetite}]+2{\text{H}}_{2}\text{O}+{\text{H}}_{2},$$

where [Fe(OH)_2_]_brucite_ represents the Fe component of brucite. As the serpentinization reaction progresses and H_2_ accumulates in solution, equilibration of this reaction should, therefore, favour higher Fe contents in brucite and limit the amount of Fe available to precipitate as magnetite. This may help explain, in part, the greater Fe contents of brucite and relatively smaller amounts of magnetite in the higher pH experiments, since these experiments accumulated higher concentrations of H_2_ (table 3). However, Rxn. 1 would also predict that magnetite production should decrease with increasing reaction progress as H_2_ accumulates, but other experimental studies of olivine serpentinization have found that the amount of magnetite produced increased at a steady rate as the reactions progressed [12,13]. Consequently, the role of thermodynamic factors in regulating the distribution of Fe between magnetite and brucite remains uncertain.

Marcaillou *et al*. [45] observed that the Fe^III ^: Fe_total_ ratio of serpentine (lizardite) increased steadily with increasing reaction progress during the serpentinization of lherzolite at 300°C. The greater partitioning of Fe^III^ into serpentine with increasing extent of reaction in their experiment was unrelated to alkaline pH, since the fluid was mildly acidic throughout the reaction (pH_25°C_ 5.1–5.8). Nevertheless, the olivine experiments in the present study appear to display a similar correspondence between higher Fe^III ^: Fe_total_ ratio with a greater extent of reaction, suggesting that increased partitioning of Fe^III^ into serpentine with increasing reaction progress may occur over a broad range of conditions.

### (b) Effect of pH on reaction of olivine–pyroxene mixtures

The increase in pH during the experiment OlivOpx230pH also led to substantially increased rates of reaction and H_2_ generation. The OlivOpx230med experiment provides some insight into the state of OlivOpx230pH prior to the pH increase. After 2709 h of heating at 230°C, the reaction of the olivine–Opx mixture in OlivOpx230med resulted in only a very limited extent of reaction (approx. 1.9 wt% of reactants) and virtually no H_2_ production [14] (table 3). Reaction products in that experiment were limited to a thin alteration rind only a few micrometre thick deposited on the surfaces of the original reactant minerals and composed of an intimate mixture of poorly crystalline lizardite and talc. Similar reaction products were likely present in OlivOpx230pH at the time that the fluid was injected to increase pH at 4526 h, although the extent of reaction may have been somewhat greater owing to the longer reaction time.

Comparison with OlivOpx230med suggests that about 4 wt% of the primary minerals in OlivOpx230pH had reacted prior to raising the pH while the extent of reaction had increased to 19 wt% by the end of the experiment at 7715 h, indicating a greater than fivefold increase in overall reaction rate following the pH increase. In both OlivOpx230pH and OlivOpx230med, the olivine : Opx ratio of the reactants that remained at the end of the experiments was much higher than in the starting materials (electronic supplementary material, figure S1), indicating reaction of Opx proceeded more rapidly than olivine. At present, however, there is not sufficient data to determine whether the increase in pH accelerated the reaction of both minerals equally or not.

**Figure 6.**
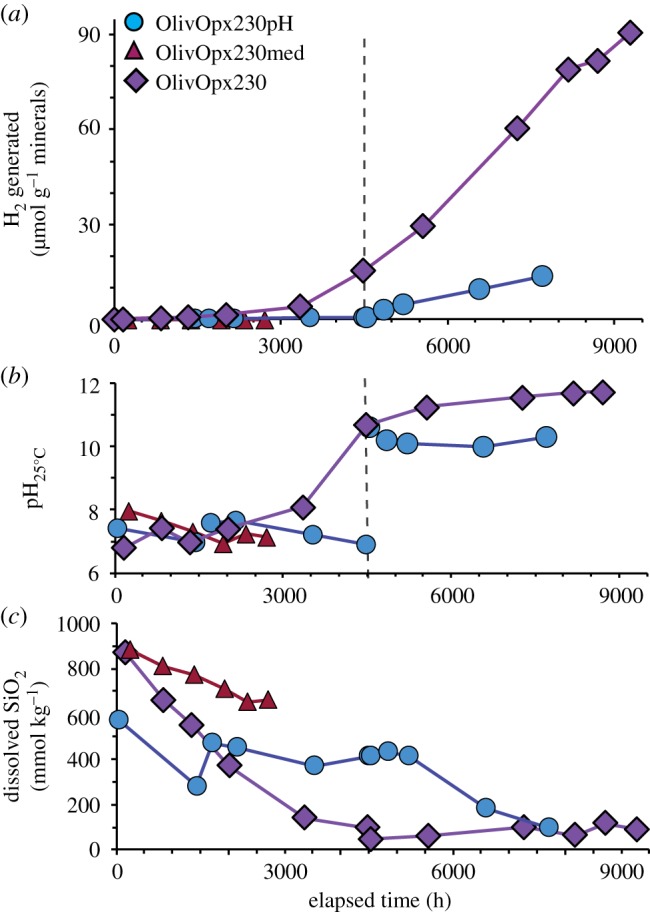
Comparison of (*a*) H_2_ production, (*b*) room temperature pH and (*c*) total dissolved SiO_2_ concentration during experiments with olivine–Opx mixtures at 230°C. The dashed vertical line denotes the timing of the injection of alkaline fluids to increase pH in the experiment OlivOpx230pH. Data for OlivOpx230 and OlivOpx230med from McCollom *et al*. [14]. (Online version in colour.)

The results of OlivOpx230pH can also be compared with those of OlivOpx230 reported by McCollom *et al*. [14]. The composition of reactant minerals and experimental conditions for OlivOpx230 were very similar to both OlivOpx230pH and OlivOpx230med (table 1), and OlivOpx230 was allowed to react for a period comparable to OlivOpx230pH. Unlike the other experiments, however, OlivOpx230 spontaneously transitioned from circumneutral to strongly alkaline conditions after approximately 4000 h of reaction without any external intervention (figure 6*b*). The reason why the pH spontaneously increased during experiment OlivOpx230 but not in the other experiments is undetermined at this time, but it appears to be related to a small amount of clinopyroxene present as a contaminant in the starting minerals for that experiment [14].

As in the experiment OlivOpx230pH, the increase in pH in OlivOpx230 was accompanied by a steep increase in H_2_ production (figure 6*a*). The overall extent of reaction and rate of H_2_ generation was significantly greater in OlivOpx230 than in OlivOpx230pH, but are comparable to the extent of reaction and H_2_ generation observed in the olivine-only experiment Oliv230pH at high pH (table 3). Reaction products of OlivOpx230 included substantial amounts of brucite and magnetite in addition to chrysotile, which likely reflects the domination of the products by the dissolution of olivine during the latter stages of the experiment [14]. No Opx remained at the end of the experiment OlivOpx230.

The spontaneous increase in pH in experiment OlivOpx230 clearly caused similar increases in rates of overall reaction and H_2_ generation to those observed following the induced increase in pH during OlivOpx230pH (figure 6*a*; table 3). More rapid reaction rates were attained in OlivOpx230 than OlivOpx230pH, which might be attributable to somewhat more strongly alkaline conditions in the former experiment (figure 6*b*) or to lower concentrations of ΣSiO_2_ (figure 6*c*). Experiment OlivOpx230 also apparently transitioned to a state where olivine dominated the overall reaction leading to precipitation of brucite and magnetite in addition to chrysotile [14], which may have contributed to greater rates of H_2_ generation.

### (c) Possible mechanism for increased rates

The observed increased rates of serpentinization and H_2_ generation at higher pH contrast with laboratory studies of olivine and pyroxene dissolution where rates have been found to decrease monotonically with increasing pH, at least up to 150°C and pH 12 [29–32]. Moreover, dissolution rates of olivine and pyroxene observed in laboratory experiments are much faster than the rates of serpentinization observed in the present study. For example, using the equation for the dissolution of olivine as a function of pH and temperature from Rimstidt *et al*. [31] based on laboratory experiments (their eqn (6)), only about 3 days should have been required to dissolve 90 wt% of the original olivine in Oliv200pH and less than 8 h for Oliv230pH, far shorter than the duration of either experiment (see electronic supplemental material for calculations). Although the rate equation of Rimstidt *et al*. is only calibrated to 150°C and ambient pressure, there is no reason to believe that rates should be slower at the higher temperatures and pressures of the present serpentinization experiments. Extrapolation of enstatite dissolution rates from laboratory studies [31] to the temperature of OlivOpx230pH would also predict that Opx should have completely dissolved on a much shorter timescale than the experiment. Evidently, feedbacks from the solution prevented the olivine and Opx in the serpentinization experiments from dissolving at the rates observed in the far-from-equilibrium laboratory dissolution studies.

While the reaction rates of olivine in the serpentinization experiments are substantially slower than dissolution studies would imply, the mechanism of dissolution determined from those studies may provide some insight into the cause for the increased rates of serpentinization at higher pH relative to those observed at circumneutral conditions. During the steady-state dissolution of olivine at acidic to slightly alkaline pH, a very thin layer (∼a few unit-cells thick) forms at the olivine surface that is enriched in Si and depleted in Mg [29,30]. At pH greater than approximately 9, however, the dissolution mechanism shifts, and a Si-depleted, Mg-enriched surface layer develops. The structure of this thin Mg-rich layer apparently resembles the individual sheets of brucite [29]. The rate-limiting step for dissolution at higher pH is thought to involve hydrolysis and detachment of Mg ions from this Si-depleted surface layer [29,30].

A similar Mg-enriched, Si-depleted layer may develop on the surface of olivine during the reaction at the higher temperatures of the serpentinization experiments. Higher pH may then increase the rate of the detachment of Mg from the surface of the Si-depleted layer, leading to faster overall reaction rates of the olivine. When brucite is present, it may enhance the dissolution rate at higher pH by lowering the concentration of Mg and continuously removing it from the solution. That is, for precipitation of brucite from solution according to the reaction (written for the pure Mg endmember)

4.2 $$\text{M}{\text{g}}^{2+}+2\text{O}{\text{H}}^{-}↔\text{ Mg}{\left(\text{OH}\right)}_{2}\;[\text{brucite}],$$

the increased concentration of OH^−^ at higher pH results in a decreased concentration of Mg in the fluid in equilibrium with brucite. Although Mg^2+^ and OH^−^ would be rapidly removed from solution by brucite precipitation, they would be continuously replenished by olivine dissolution

4.3 $$\text{M}{\text{g}}_{2}\text{Si}{\text{O}}_{4}\;[\text{olivine}]+2{\text{H}}_{2}\text{O }\to \text{ Si}{\text{O}}_{2(\text{aq})}+2\text{M}{\text{g}}^{2+}+4\text{O}{\text{H}}^{-}.$$

Notably, the Si-depleted layer forms on dissolving olivine even when the solution is undersaturated with respect to brucite [29,30], so this layer may also be present at the olivine surface in the mixed olivine–Opx experiments at higher pH [14].

**Figure 7.**
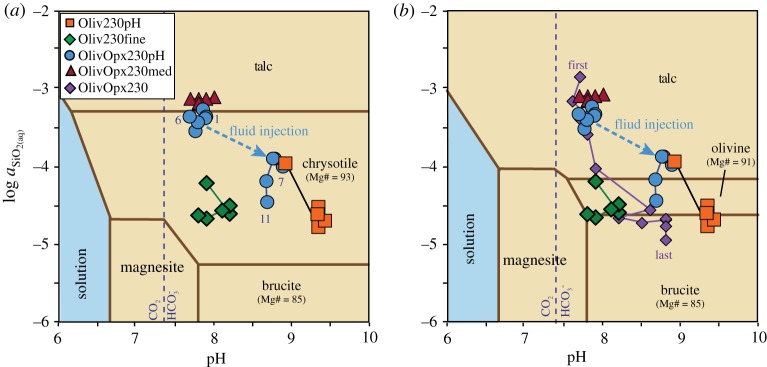
Calculated *in situ* fluid compositions for laboratory experiments projected onto mineral stability diagrams for Mg-bearing minerals in the Mg–Fe–Si–O–C–H system as a function of pH and activity of dissolved silica. (*a*) shows stable equilibrium mineral assemblages at 230°C, while (*b*) shows metastable assemblages calculated by excluding equilibration with chrysotile. Numbers in (*a*) identify sample numbers for OlivOpx230pH and in (*b*) the first and last samples for OlivOpx230 are identified. Compositions of chrysotile and brucite are constrained to approximate those from the experiments. For the construction of the diagrams, the activity of dissolved Mg was set to 10^−5^, H_2_ to 10^−2^ and $\text{HC}{{\text{O}}_{3}}^{-}$ to 10^−2.5^, while dissolved Fe was assumed to in equilibrium with magnetite. Dashed vertical lines separate predominance fields for CO_2(aq)_ and $\text{HC}{{\text{O}}_{3}}^{-}$ for dissolved carbon species. (Online version in colour.)

The concentrations of ΣSiO_2_ in the fluids provides support for the formation of an Mg-rich layer on the olivine surface during the experiments. Figure 7*a* shows the calculated activities of dissolved silica [*a*_SiO_2_(aq)_] and pH*_in situ_* during the 230°C experiments projected onto an equilibrium mineral stability diagram for the experimental conditions. It can be seen from the figure that the fluid compositions for the olivine-only experiments plot within the chrysotile stability field and well above the chrysotile–brucite boundary, despite the presence of abundant brucite in both experiments. Under these conditions, brucite should react with dissolved SiO_2_ to form chrysotile, lowering the *a*_SiO_2_(aq)_ to the chrysotile–brucite equilibrium boundary. The persistence of *a*_SiO_2_(aq)_ well above this level probably indicates kinetic inhibition of the reaction between brucite and dissolved SiO_2_ [23], precluding equilibrium between brucite and chrysotile.

Instead, it appears that the activity of dissolved silica in the experiments may be controlled by steady-state reactions near the brucite–olivine equilibrium boundary. Figure 7*b* shows a mineral stability diagram where equilibrium with chrysotile has been suppressed. As seen in the diagram, the fluid compositions during the olivine experiments converge on the olivine–brucite boundary, suggesting that *a*_SiO_2_(aq)_ levels are being regulated to values near equilibrium between olivine and brucite (or a brucite-like phase). In this case, this would represent a metastable equilibrium, since olivine is thermodynamically unstable and would be replaced by chrysotile at full equilibrium. Prior to the increase in pH in the experiment OlivOpx230pH, the *a*_SiO_2_(aq)_ was apparently buffered at relatively high levels by chrysotile–talc equilibrium (figure 7*a*). However, following the pH increase the *a*_SiO_2_(aq)_ in this experiment also converged on the olivine–brucite equilibrium boundary towards the end of the experiment (figure 7*b*). Silica activities consistent with metastable olivine–brucite equilibrium have also been observed in several other serpentinization experiments [13,14], as illustrated by the results for Oliv230fine and OlivOpx230 shown in figure 7*b*.

It may be that the dissolved silica activities in these experiments are being regulated by steady-state dissolution and interactions between the Mg-enriched surface layer and the underlying olivine [14]. At the interface between the surface layer and olivine, the exchange of SiO_2_ with the fluid may be rapid and maintain a local metastable equilibrium between the mineral surface and dissolved silica in the fluid. Given the apparent brucite-like structure of the Mg-enriched layer, the resulting metastable equilibrium between the surface layer and underlying olivine would be expected to occur at a silica activity close to brucite–olivine equilibrium.

It should be noted that although the silica activities converge on similar values in these experiments, that does not necessarily mean that the experiments will have similar concentrations of total dissolved silica since the speciation varies substantially as a function of pH. As shown in electronic supplementary material, figure S4, at pH*_in situ_* less than 8.4 the speciation of dissolved silica is dominated by SiO_2(aq)_, but as the pH*_in situ_* rises above 8.4 the speciation is increasingly dominated by $\text{HSi}{{\text{O}}_{3}}^{-}$ and possibly NaHSiO_3(aq)_, although the stability of the latter under hydrothermal conditions is currently uncertain [46]. As a consequence, at constant *a*_SiO_2_(aq)_ the total dissolved silica concentration (i.e. ΣSiO_2_) increases steeply with increasing pH*_in situ_* above 8.4 (electronic supplementary material, figure S4). This may help explain why the ΣSiO_2_ concentrations in the higher pH experiments are substantially higher than those in the lower pH experiments (figures 1*c* and 4*c*).

The elevated ΣSiO_2_ concentrations in the higher pH experiments may also contribute to the more rapid reactions rates observed for those experiments. The elevated ΣSiO_2_ concentrations may promote more rapid precipitation of chrysotile and therefore the dissolution of olivine, leading to more rapid overall reaction relative to less alkaline pH conditions where ΣSiO_2_ concentrations are lower. In addition, the steep increase in ΣSiO_2_ concentrations at increasingly alkaline conditions may help regulate the pH in the experiments, since raising the pH further would require increasingly higher concentrations of ΣSiO_2_ that would be counteracted by precipitation of chrysotile.

The higher pH might also induce an increase in the precipitation rate of brucite, and thereby lead to an increase in overall reaction rate. Specifically, removal of Mg and Fe from solution owing to faster precipitation of brucite may cause these elements to be removed at a greater rate from the surface of olivine, leading to an increase in olivine dissolution rate. A detachment of Mg ions from the surface is thought to be the rate-limiting step for the dissolution of olivine at alkaline pH in laboratory dissolution studies [29,30], and the same may hold true during serpentinization.

Testing of each of these possibilities will require additional experimental studies that continue to probe the effect of different fluid parameters on rates of individual reactions taking place during serpentinization (e.g. [23]). However, regardless of the underlying cause, the experiments in this study indicate that the strongly alkaline pH that develops in many serpentinizing systems will result in a steep increase in overall reaction rates. Because this outcome is contrary to expectations based on lower temperature dissolution studies, further study and elucidation of the underlying mechanisms is warranted in order to better understand serpentinization in natural systems.

### (d) Implications for natural systems

The results of this study suggest that higher pH fluids circulating through ultramafic rocks are likely to induce more rapid serpentinization and higher H_2_ fluxes than lower pH fluids. In subseafloor settings, for example, this may result in a greater extent of serpentinization of the rocks and higher concentrations of H_2_ discharged at the seafloor in hydrothermal fluids. As seawater begins to circulate through seafloor hydrothermal systems, it has a slightly alkaline pH (≈8) and any interaction of the fluid with ultramafic rocks may initially result in modest rates of reaction and H_2_ generation. However, as the circulating fluid evolves through water–rock interactions and transitions to strongly alkaline pH (as in OlivOpx230; figure 6), rates of reaction and H_2_ generation would be expected to accelerate. Other factors such as the rate of fluid circulation, bulk rock composition and temperature will undoubtedly influence the amount of H_2_ that is generated as well, but the experimental results indicate that the evolving pH along the flow path of circulating fluids may be a critical factor in regulating the flux of H_2_ discharged from actively serpentinizing systems.

The experimental results also indicate that pH affects Fe partitioning among the mineral products during serpentinization. Serpentinized rocks recovered from the ocean floor and from ophiolites exhibit substantial variation in the Fe contents of chrysotile and brucite and in the amount of magnetite present (e.g. [47–55]), but the underlying factors that regulate these variations remain incompletely understood. The results of this study indicate that pH needs to be considered along with other factors such as temperature, rock composition and silica activity in interpreting the distribution of Fe in serpentinized rocks. If the experiments from this study are representative, the results suggest that higher pH will lead to increased partitioning of Fe into serpentine and brucite rather than magnetite, as well as greater incorporation of Fe^III^ in serpentine. However, further study is required to confirm these trends and more confidently determine the role of pH in the distribution of Fe during serpentinization.

## Supplementary Material

Supplemental Materials

## Data accessibility

Supporting data for the figures are included in the electronic supplementary material.
