# Supplementary material for: The effect of pH on rates of reaction and hydrogen generation during serpentinization
Source: Philos Trans A Math Phys Eng Sci. 2020 Jan 6;378(2165):20180428. doi: 10.1098/rsta.2018.0428 (PMC7015308; doi:10.1098/rsta.2018.0428)
Supplement: Supplemental Materials [file rsta20180428supp1.docx]

The Effect of pH on Rates of Reaction and Hydrogen Generation During Serpentinization

Thomas M. McCollom^1^, Frieder Klein^2^, Peter Solheid^3,4^, Bruce Moskowitz^3,4^

# **Additional methods**

Analyses of dissolved H_2_, total dissolved CO_2_, CH_4_, and C_2_-C_6_ hydrocarbons were performed using procedures described in detail in McCollom et al. (2001) and McCollom and Seewald (2001). Briefly, concentrations of total dissolved CO_2_ (ΣCO_2_ = CO_2_*_(aq)_* + HCO_3_^-^ + CO_3_^2-^) were determined by gas chromatography (GC) with thermoconductivity detection (TCD) following acidification of the sample with 25% phosphoric acid and extraction into a headspace with 1 ml He gas. Measured amounts of ΣCO_2_ were corrected to account for the fraction remaining dissolved in the acidified fluid. Methane and hydrocarbons were analysed on the same sample using Flame Ionization Detection (FID). Dissolved H_2_ was analysed by GC with thermoconductivity detection (TCD) following a headspace extraction into 1 ml N_2_.

To account for differences in fluid:rock ratios between experiments and changing amounts of fluid in the reaction cell over time as a result of sampling, the amount of H_2_ generated was normalized by calculating the total number of moles of H_2_ generated per gram of mineral reactants. For the calculations, the total amount of H_2_ generated during each sample interval was determined by multiplying the change in dissolved H_2_ concentration by the amount of fluid present in the reaction vessel at the time of sampling, and then dividing by the initial mass of minerals included in the experiments. To simplify the calculation, the small amounts of water incorporated into product minerals or converted to H_2_ were neglected.

The pH*_25°C_* was measured using a Ross micro combination electrode at room temperature with an uncertainty of approximately ±0.1 units of the reported value. Dissolved SiO_2_ was measured immediately after sampling with a Hach DR/2400 spectrophotometer using the heteropoly-blue method at a wavelength of 815 nm, with reagents and methods supplied by the manufacturer. An aliquot of the fluid was retained for analysis of major elements after dilution and acidification with a small amount of HNO_3_, and subsequently analysed for total dissolved Na, Ca, Mg, Fe, Si, and K by Inductively Coupled Plasma – Optical Emission Spectroscopy (ICP-OES). In almost all cases, the Si concentrations measured by ICP-OES were within 10% of SiO_2_ values determined by spectrophotometry, so only the latter are reported here. At selected time points, fluid aliquots were analysed for total dissolved chloride using titration against silver nitrate with chromate as an indicator.

Initial characterization of the morphology and chemical composition of solid reaction products was performed using a JEOL 6480LV Scanning Electron Microscope (SEM) equipped with an Oxford Instruments Electron-dispersive X-Ray Spectrometer (EDS) and INCA data processing software. Solids for SEM/EDS were examined both as uncoated grains mounted on Al stubs with carbon tape and as polished grain mounts embedded in epoxy and coated with carbon, using an accelerating voltage of 15 kV. Quantitative compositional data were obtained with Electron Microprobe Analysis (EMPA) using a JEOL JXA 8600 at the University of Colorado, which is equipped with five wavelength-dispersive spectrometers. Minerals were analysed with an accelerating voltage of 15 kV for a beam current of 15–20 nA and a defocused 5 μm beam diameter. Owing to the porous nature of the mats of chrysotile fibres that formed in the experiments, the element totals for the analyses were typically significantly lower (60-77%) than would be expected for ideal chrysotile (87%). Raw data were corrected using the CITZAF method.

Raman spectra of solid reaction products were obtained using a Horiba LabRAM HR confocal spectrometer equipped with a 17 mW 633 nm HeNe laser, an astigmatic flat field spectrograph with a focal length of 800 mm, and a multichannel air-cooled (-70 °C) CCD detector. Individual spectra were recorded using a 40× long-distance objective. A grating with 600 grooves / mm and a confocal hole diameter of 100 to 200 µm was chosen for most analyses. Spectra were collected for 5 seconds with 3 accumulations between 100 cm^-1^ and 1300 cm^-1^ and between 3500 cm^-1^ and 3800 cm^-1^.

Mössbauer spectroscopy and magnetic investigations were carried out on powdered samples (~50-100 mg) at the Institute for Rock Magnetism (IRM), University of Minnesota, USA. Mössbauer spectra were measured at room temperature and in zero applied field using a conventional constant-acceleration spectrometer (SeeCo) in transmission geometry with a ^57^Co/Rh source. An α-Fe foil at room temperature was used to calibrate isomer shifts and velocity scale. Mössbauer spectra were fit using custom software to obtain estimates for isomer shift (IS), quadrupole splitting (QS), linewidth (Γ), and relative area (A) for each doublet subspectrum. To focus on secondary products (primarily Fe in serpentine), secondary products were physically separated from the bulk solids (including remnant primary minerals) by sonication with ethanol followed by removal of the suspended fraction. This procedure was repeated until the ethanol was no longer cloudy, and Mössbauer analysis performed on this prepared sample following evaporation of the ethanol. Magnetite was removed from the reacted solids during this process using a strong magnet. Inspection of the remnant solids by SEM indicated that this method was effective in separating nearly all of the secondary products from unreacted primary minerals. No magnetically-ordered sextets (e.g., associated with magnetite or hematite) were observed in any of the samples so only doublets were used for fitting spectra.

Room temperature hysteresis loops were obtained in a vibrating sample magnetometer (Princeton Measurements Corporation) using an electromagnet to produce fields up to 1 T. Hysteresis parameters (saturation magnetization, M_s_; saturation remanence, M_r_; coercivity, B_c_) were calculated after paramagnetic slope correction over the interval of 0.7-1.0 T using the non-linear approach-to-saturation fitting method of Jackson and Solheid [2010]. The magnetite weight-percent was determined from the room-temperature bulk saturation magnetization values and the known value for pure magnetite (M_s_=92 Am^2^/kg) as follows:

Magnetite weight percent = $\frac{\text{M}_{\text{s}}(sample)}{\text{M}_{\text{s}}(magnetite)}\text{ × 100 percent}$.

Thermogravimetric analyses (TGA) were performed on the reacted solids to determine the amount of hydrated minerals present, and these data were then used to estimate the amount of primary minerals reacted. The analyses were performed using a Thermal Analysis (TA) Instruments (Lindon, UT) SDT Q600 simultaneous thermogravimetric analyzer-differential scanning calorimeter at Woods Hole Oceanographic Institution. Finely ground samples (~30-50 mg) were weighed into an alumina crucible and heated from room temperature to 1100 °C at 10 °C per minute while monitoring the change in mass and heat flow.  An empty crucible was measured as a blank value simultaneous with the sample to minimize errors in sample weighing during heating. Nitrogen (N_2_) was used as the purge gas at a flow rate of 50 ml per minute. The results show peak weight loss corresponding to dehydration of brucite at 320-370 °C, and of chrysotile at 600-650 °C^1^.

# **Calculation of olivine dissolution rates at experimental conditions**

For pH > 5.6, Rimstidt et al. (2012) proposed that the rate of olivine dissolution can be represented by the equation:

log *r_geo_* = 4.07 – 0.256pH -3465/*T*

where *r_geo_* is the dissolution rate of olivine based on geometric surface area in units of mol m^-2^ s^-1^ and *T* is temperature in Kelvin. For an in situ pH of 8.6 in Oliv200pH and 9.3 in Oliv230pH, calculated values of *r_geo_* are 3.2 × 10^-6^ mol m^-2^ s^-1^ and 6.3 × 10^-6^ mol m^-2^ s^-1^, respectively. Assuming geometric surface areas of 0.032 and 0.129 m^2^ g^-1^ in the experiments and a molecular weight for olivine of 146 g mol^-1^, estimated dissolution rates are 1.6 × 10^-5^ (g dissolved) (g olivine)^-1^ s^-1^ for Oliv200pH and 1.2 × 10^-4^ (g dissolved) (g olivine)^-1^ s^-1^ for Oliv230pH.


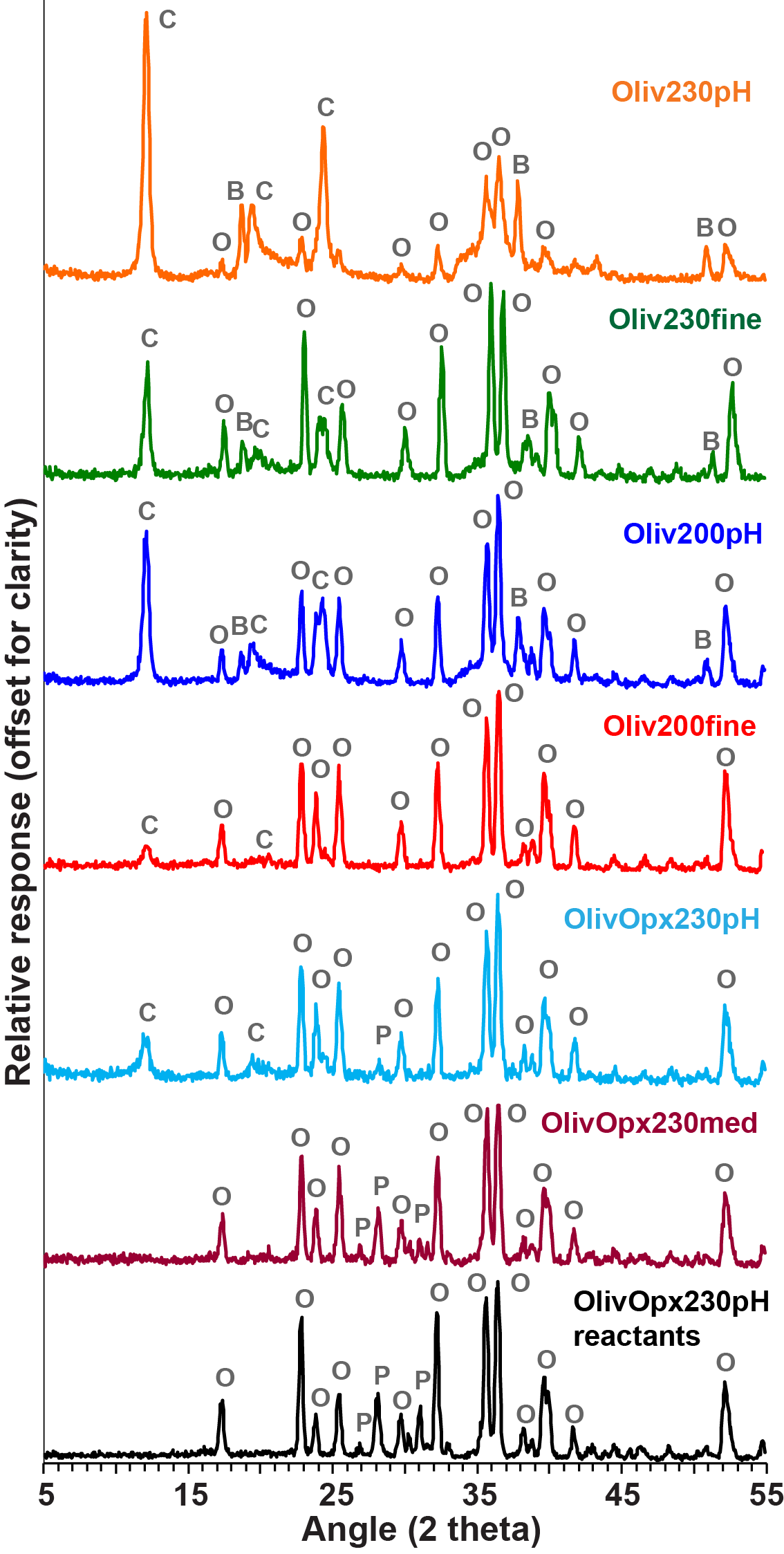


**Supplementary Figure S1:** Results of X-ray diffraction analysis of reaction products and starting materials. Mineral abbreviations: C = chrysotile, B = brucite, O = olivine, P = orthopyroxene.


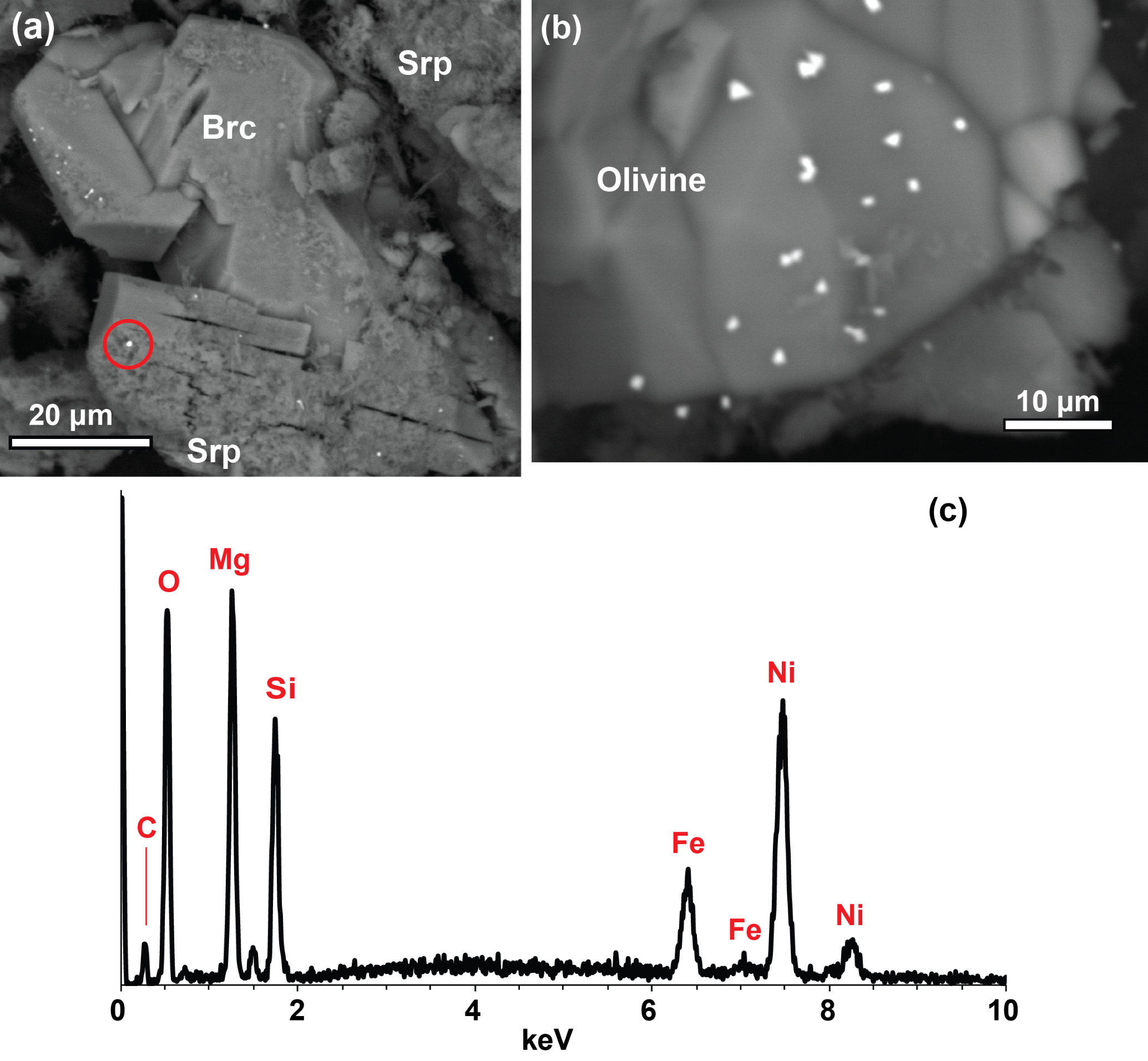


**Supplementary Figure S2:** Particles enriched in Ni and Fe from experiment Oliv230pH. (a) Back-scattered electron images of reaction products, displaying small (<1 µm) bright particles enriched in Ni and Fe. (c) EDS spectrum of bright particle shown outlined by circle in (a). The Mg and Si in the spectrum derive from chrysotile crystals surrounding the particles. The lack of S and O enrichments indicate the Ni and Fe are likely a native a Ni-Fe alloy such as awaruite (the amount of O present in the spectrum is consistent with chrysotile elsewhere in sample, and would be expected to be much larger if the Ni-Fe was present as an oxide). The Ni-Fe particles were generally found in close proximity to partially reacted olivine and secondary brucite. Brc = brucite, Srp = chrysotile serpentine.

**Supplementary Figure S3:** Mössbauer spectra and fits measured at 300 K. Data shown by individual symbols. Best fit is solid black line and individual doublets in colored lines. D1 is Fe^II^, D2 is Fe^III^ (octahedral), D3 is Fe^III^ (tetrahedral), and D4 is Fe^II^


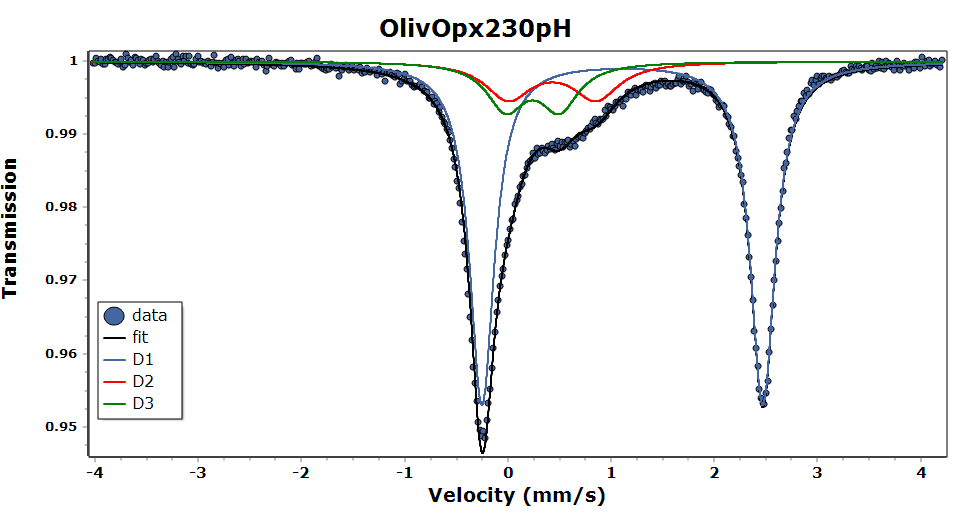

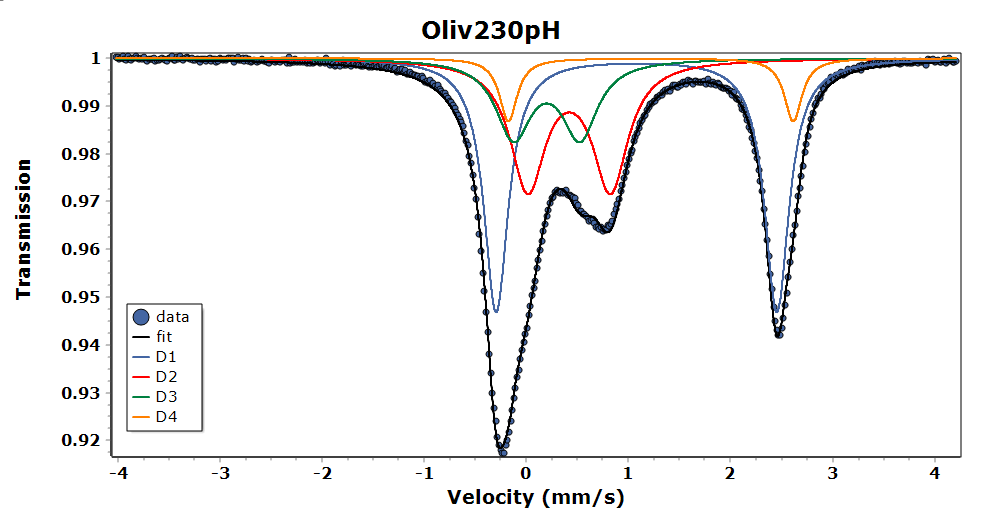

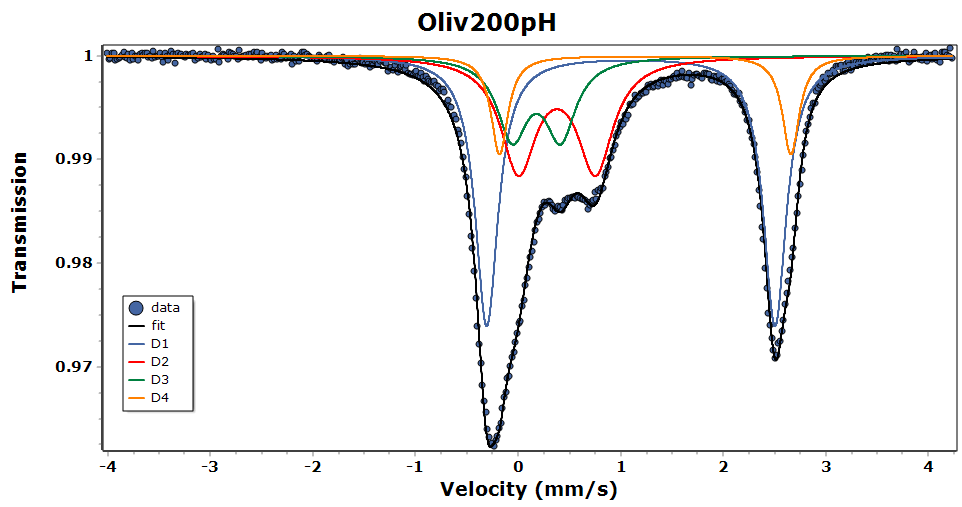


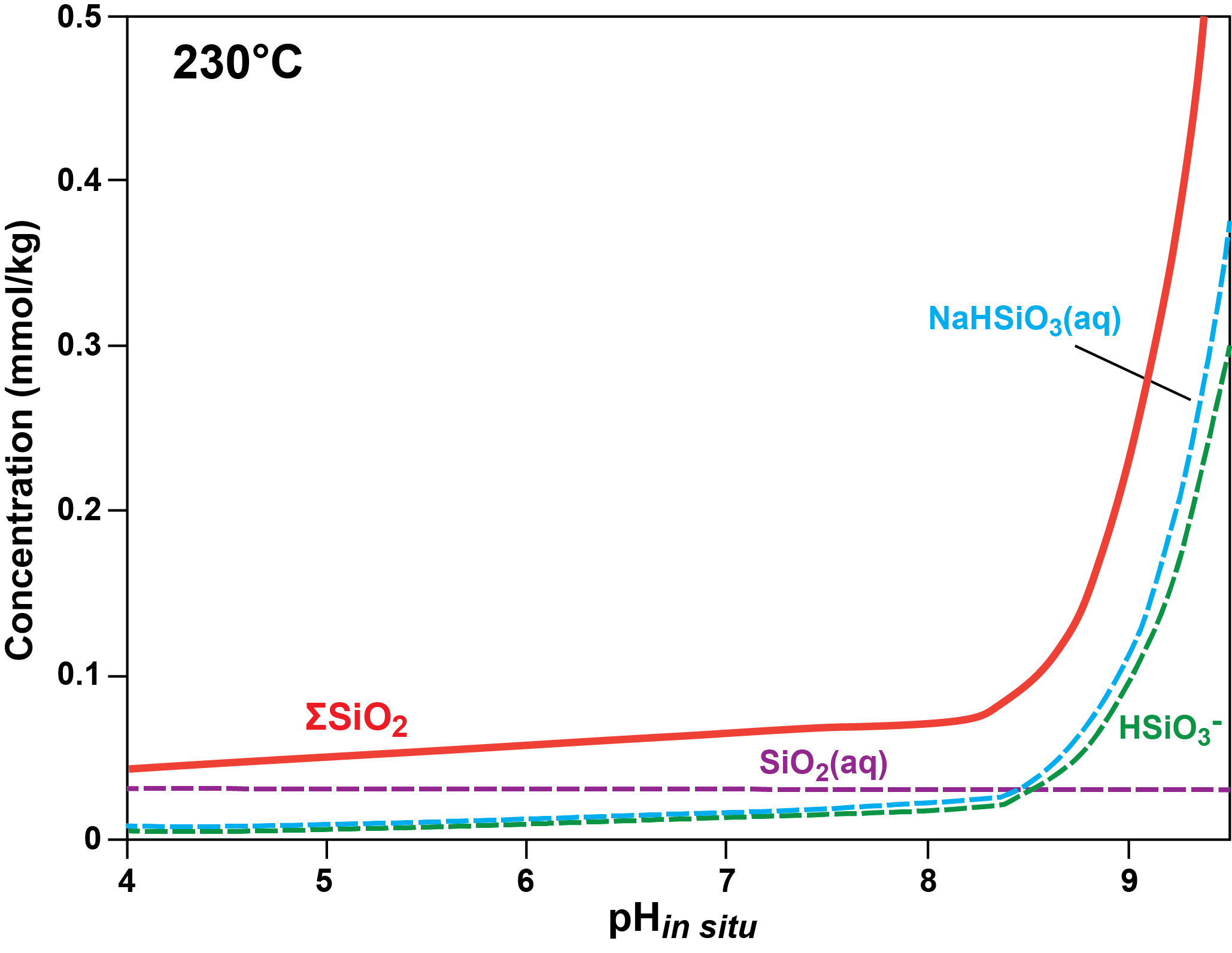


**Supplementary Figure S4:** Speciation of total dissolved SiO_2_ as a function of in situ pH at a constant *a*_SiO2_*_(aq)_* = 10^-4.5^, temperature = 230°C, and pressure = 35 MPa. Diagram is drawn for Cl = 485 mmol/kg and Na concentration varied to achieve charge balance. Speciation calculations performed with Geochemist’s Workbench using the thermodynamic database of McCollom and Bach (2009).

**Supplementary Table S1:** Fluid compositions during experiments.

| Time *(h)* | Temp *(°C)* | H_2_ *(m)* | | CO_2_ *(m)* | | CH_4_ *(μ)* | | Na *(m)* | Cl *(m)* | | Si *(u)* | | | Fe *(μ)* | | Mg *(μ)* | | Ca *(μ)* | | Al *(μ)* | | K *(μ)* | | pH (25ºC) | | pH in situ | H_2_ gen.^#^ *(µmol/g)* | Fluid *(g)* |
| --- | --- | --- | --- | --- | --- | --- | --- | --- | --- | --- | --- | --- | --- | --- | --- | --- | --- | --- | --- | --- | --- | --- | --- | --- | --- | --- | --- | --- |
| ***Experiment Oliv230pH (****230 ºC)* | | | | | | | | | | | | | | | | | | | | | | | | | | | | |
| 45 | 230 | 0.06 | | 3.4 | | 17 | | 480 | | - | | | 480 | | b.d. | | b.d. | | b.d. | | b.d. | | 6400 | | 11.8 | 8.9 | 0.18 | 45.7 |
| 333 | 230 | 8.6 | | 2.1 | | 19 | | 505 | | - | | | 180 | | b.d. | | b.d. | | b.d. | | 67 | | 7500 | | 12.3 | 9.3 | 24 | 41.4 |
| 669 | 230 | 16.4 | | 1.7 | | 20 | | 480 | | - | | | 320 | | b.d. | | b.d. | | b.d. | | b.d. | | 7300 | | 12.3 | 9.3 | 43 | 37.1 |
| 1462 | 230 | 33. | | 0.8 | | 21 | | 480 | | - | | | 250 | | b.d. | | 15 | | b.d. | | b.d. | | 7300 | | 12.4 | 9.4 | 79 | 32.5 |
| 2972 | 230 | 47. | | 0.4 | | 22 | | 490 | | 470 | | | 240 | | b.d. | | 13 | | b.d. | | b.d. | | 6700 | | 12.3 | 9.3 | 105 | 29.2 |
| ***Experiment Oliv200pH (****200 ºC)* | | | | | | | | | | | | | | | | | | | | | | | | | | | | |
| 46 | 202 | 0.0076 | | 2.6 | | 7 | | 526 | | - | | | 430 | | b.d. | | b.d. | | b.d. | | 90 | | 810 | | 12.4 | 9.6 | 0.028 | 51.2 |
| 502 | 202 | 0.71 | | 2.5 | | 8 | | 510 | | - | | | 200 | | b.d. | | b.d. | | b.d. | | b.d. | | 500 | | 12.4 | 9.6 | 2.3 | 46.9 |
| 1342 | 200 | 0.69 | | 2.3 | | 8 | | 490 | | - | | | 190 | | b.d. | | b.d. | | b.d. | | b.d. | | 1030 | | 12.4 | 9.6 | 2.3 | 43.2 |
| 2541 | 200 | 0.64 | | - | | - | | - | | - | | | 130 | | - | | - | | - | | - | | - | | 12.0 | 9.2 | 2.2 | 39.2 |
| 4560 | 200 | 2.66 | | 1.7 | | 9 | | 405 | | 470 | | | 77 | | b.d. | | 10 | | 50 | | 70 | | 960 | | 11.4 | 8.7 | 7.5 | 37.5 |
| 7677 | 200 | 8.40 | | 0.8 | | 9 | | 455 | | 460 | | | 63 | | b.d. | | b.d. | | 70 | | b.d. | | 950 | | 11.3 | 8.5 | 21. | 32.8 |
| ***Experiment OlivOpx230pH (****230 ºC)* | | | | | | | | | | | | | | | | | | | | | | | | | | | | |
| 52 | 231 | | <0.001 | | 20.5 | | 58 | 513 | | - | | 570 | | b.d. | | 100 | | 70 | | 60 | | 40 | | 7.4 | | 7.9 | b.d. | 35.8 |
| 1454 | 231 | | 0.02 | | 21.9 | | 47 | - | | 480 | | 280 | | - | | - | | - | | - | | - | | 7.0 | | 7.8 | 0.05 | 31.8 |
| 1530 | *–– Injected 10 g fluid containing 432 mmol kg^-1^ NaCl, 18 mmol kg^-1^ Na_2_CO_3_, and 3 mmol kg^-1^ NaOH; pH =10.6 ––* | | | | | | | | | | | | | | | | | | | | | | | | | | | |
| 1718 | 231 | | 0.04 | | - | | - | 482 | | - | | 470 | | b.d. | | 70 | | 60 | | 50 | | 220 | | 7.6 | | 7.9 | 0.11 | 40.9 |
| 2173 | 231 | | 0.13 | | 18.0 | | 22 | 506 | | - | | 450 | | b.d. | | b.d. | | 40 | | 80 | | 220 | | 7.7 | | 7.9 | 0.31 | 37.5 |
| 3540 | 231 | | 0.20 | | 16.4 | | - | 461 | | - | | 370 | | b.d. | | b.d. | | 60 | | 70 | | 160 | | 7.2 | | 7.8 | 0.45 | 34.3 |
| 4503 | 230 | | 0.26 | | 17.2 | | 9 | 469 | | 460 | | 410 | | b.d. | | b.d. | | 70 | | b.d. | | 240 | | 6.9 | | 7.7 | 0.55 | 30.0 |
| 4526 | *–– Injected 14 g fluid containing 480 mmol kg^-1^ NaCl, 18 mmol kg^-1^ Na_2_CO_3_, and 41 mmol kg^-1^ NaOH; pH =12.3 ––* | | | | | | | | | | | | | | | | | | | | | | | | | | | |
| 4553 | 230 | | 0.15 | | 19.0 | | 35 | - | | - | | 410 | | b.d. | | b.d. | | - | | - | | - | | 10.6 | | 8.9 | 0.59 | 40.8 |
| 4863 | 230 | | 1.27 | | 18.2 | | 7 | 505 | | - | | 430 | | b.d. | | b.d. | | 50 | | b.d. | | b.d. | | 10.2 | | 8.8 | 3.1 | 37.4 |
| 5227 | 230 | | 2.09 | | 18.3 | | 7 | 501 | | - | | 410 | | b.d. | | b.d | | 60 | | b.d. | | b.d. | | 10.1 | | 8.8 | 4.7 | 33.1 |
| 6594 | 230 | | 4.79 | | 15.8 | | 9 | 494 | | - | | 180 | | b.d. | | b.d. | | 30 | | b.d. | | b.d. | | 10.0 | | 8.7 | 9.6 | 30.0 |
| 7715 | 230 | | 7.35 | | 13.0 | | 10 | 501 | | - | | 100 | | b.d. | | b.d. | | 50 | | b.d. | | b.d. | | 10.3 | | 8.7 | 13.5 | 25.8 |

Concentrations in *m*mol kg^-1^ (*m*) or μmol kg^-1^ (*μ*). Concentrations given as “b.d.” were below the detection limit of approximately 3 μmol/kg. “-“ = not measured. ^#^Hydrogen generated per gram of reactant olivine. ^†^Amount of fluid in reaction cell at time of sampling.

**References**

1. Viti C. (2008) Serpentine minerals discrimination by thermal analysis. *Am. Mineral.* **95,** 631-638.
